# A family of pathogen-induced cysteine-rich transmembrane proteins is involved in plant disease resistance

**DOI:** 10.1007/s00425-021-03606-3

**Published:** 2021-04-15

**Authors:** Marciel Pereira Mendes, Richard Hickman, Marcel C. Van Verk, Nicole M. Nieuwendijk, Anja Reinstädler, Ralph Panstruga, Corné M. J. Pieterse, Saskia C. M. Van Wees

**Affiliations:** 1 Plant-Microbe Interactions, Department of Biology, Science4Life, Utrecht University, 800.56, 3508 TB Utrecht, The Netherlands; 2 Bioinformatics, Department of Biology, Science4Life, Utrecht University, 800.56, 3508 TB Utrecht, The Netherlands; 3 Institute for Biology I, Unit of Plant Molecular Cell Biology, RWTH Aachen University, Worringerweg 1, 52056 Aachen, Germany

**Keywords:** Biotrophic pathogens, Comparative genomics, Immunity, Light responses, Peptides, Salicylic acid

## Abstract

**Main conclusion:**

Overexpression of pathogen-induced cysteine-rich transmembrane proteins (PCMs) in *Arabidopsis thaliana* enhances resistance against biotrophic pathogens and stimulates hypocotyl growth, suggesting a potential role for PCMs in connecting both biological processes.

**Abstract:**

Plants possess a sophisticated immune system to protect themselves against pathogen attack. The defense hormone salicylic acid (SA) is an important player in the plant immune gene regulatory network. Using RNA-seq time series data of *Arabidopsis thaliana* leaves treated with SA, we identified a largely uncharacterized SA-responsive gene family of eight members that are all activated in response to various pathogens or their immune elicitors and encode small proteins with cysteine-rich transmembrane domains. Based on their nucleotide similarity and chromosomal position, the designated Pathogen-induced Cysteine-rich transMembrane protein (PCM) genes were subdivided into three subgroups consisting of *PCM1-3* (subgroup I), *PCM4-6* (subgroup II), and *PCM7-8* (subgroup III). Of the PCM genes, only *PCM4* (also known as *PCC1*) has previously been implicated in plant immunity. Transient expression assays in *Nicotiana benthamiana* indicated that most PCM proteins localize to the plasma membrane. Ectopic overexpression of the PCMs in *Arabidopsis thaliana* resulted in all eight cases in enhanced resistance against the biotrophic oomycete pathogen *Hyaloperonospora arabidopsidis* Noco2. Additionally, overexpression of PCM subgroup I genes conferred enhanced resistance to the hemi-biotrophic bacterial pathogen *Pseudomonas syringae* pv. *tomato* DC3000. The PCM-overexpression lines were found to be also affected in the expression of genes related to light signaling and development, and accordingly, PCM-overexpressing seedlings displayed elongated hypocotyl growth. These results point to a function of PCMs in both disease resistance and photomorphogenesis, connecting both biological processes, possibly via effects on membrane structure or activity of interacting proteins at the plasma membrane.

**Supplementary Information:**

The online version contains supplementary material available at 10.1007/s00425-021-03606-3.

## Introduction

In nature and in agriculture, plants are exposed to many different pathogenic microorganisms. To counter these threats, plants have evolved a complex immune system that can perceive pathogens and activate an appropriate response. These induced defense responses aim to fortify physical barriers against pathogen entry, such as callose (Luna et al. 2011). In addition, defensive compounds like secondary metabolites and pathogenesis-related proteins (PRs) accumulate, some of which have been demonstrated to possess in vitro antimicrobial activity and are associated with plant resistance (van Loon et al. 2006; Sels et al. 2008; Gamir et al. 2017). Plants can rely on a rich repertoire of defense compounds to combat different infecting agents. Still, many of the genes induced during pathogen infection have a so far unknown function, even though a role in defense can be expected for many of them.

The plant immune gene regulatory network that is activated in response to pathogen infection instructs which responses are expressed upon recognition of a specific invader. Conserved microbe-associated molecular patterns (MAMPs) and specific pathogen effectors can be perceived by matching receptors in the plant (Dodds and Rathjen 2010), which subsequently activate diverse downstream signaling cascades that involve elevated levels of reactive oxygen species and calcium signaling, the modification of enzymes, and changes in hormone levels (Boller and Felix 2009). The phytohormone salicylic acid (SA) plays a key role as signaling molecule in the regulation of plant immune responses that are primarily effective to fight biotrophic pathogens (Fu and Dong 2013). In SA-activated cells, the transcriptional cofactor NONEXPRESSOR OF PATHOGENESIS-RELATED GENES1 (NPR1) interacts with members of the TGA family of transcription factors, leading to transcriptional activation of different other transcription factors, like members of the WRKY family, and downstream SA-responsive defense genes (Tsuda and Somssich 2015). Microarray analysis of *Arabidopsis thaliana* (hereafter: Arabidopsis) plants expressing an NPR1-GR (glucocorticoid receptor) fusion protein (Wang et al. 2006) showed that several well-known SA-related genes, like those encoding PR proteins and WRKY transcription factors, were among the differentially expressed genes (DEGs) following SA treatment and dexamethasone-induced nuclear localization of NPR1. Almost 20% of the 64 direct target genes regulated by NPR1 were described as having an unknown or uncharacterized function.

While the role of SA in regulating responses to pathogen infection is well established, it is also known to have a broader influence, regulating responses to abiotic stresses, such as cold, heat shock, drought, high salinity, UV radiation, and shade avoidance (Hayat et al. 2010; Nozue et al. 2018). SA also impacts plant growth by inhibiting auxin (growth hormone) signaling and contributes to developmental processes, such as flower formation. The latter is delayed in SA-deficient Arabidopsis genotypes (*NahG* transgenic lines; *eds5* and *sid2* mutants), suggesting an interplay of SA with photoperiod and autonomous (flowering) pathways (Martinez et al. 2004; Rivas-San Vicente and Plasencia 2011).

Even though the complete Arabidopsis genome has been known for nearly two decades (Arabidopsis Genome Initiative 2000), a large fraction of the protein-coding genes is still lacking a meaningful functional characterization (Niehaus et al. 2015). A common starting point for gene characterization is to reveal the conditions under which a gene is expressed. Transcriptome analysis has been extensively used to pinpoint genes that are active in specific tissue/cell types, at developmental stages or in response to different stimuli. Recently, several research groups utilized time series transcriptome experiments in the model plant Arabidopsis to gain insight into the topology of the gene regulatory network that is engaged under different conditions. These experiments provided a wealth of predictions regarding functional and regulatory roles of sets of genes that are differentially expressed in diverse situations (Krouk et al. 2010; Breeze et al. 2011; Bar-Joseph et al. 2012; Windram et al. 2012; Lewis et al. 2015; Coolen et al. 2016; Hickman et al. 2017). In our recent study, we applied whole transcriptome shotgun sequencing (RNA-seq) time series and found that approximately one-third of the Arabidopsis genome was differentially expressed in leaves upon treatment with SA over a 16-h time course, with changes in gene expression occurring in well-defined process-specific waves of induction or repression (Hickman et al. 2019).

Here, this SA-responsive gene set was analyzed with the comparative genomics tools OrthoMCL and JackHMMER, which identified homologous groups of largely uncharacterized genes that may play a role in SA-associated immunity. This integrated analysis categorized over a hundred groups of SA-responsive genes, including one group of eight genes encoding short proteins that share a predicted cysteine-rich transmembrane domain and are also responsive to various pathogens and immune elicitors. We therefore named them Pathogen-induced Cysteine-rich transMembrane proteins (PCMs). The PCMs are also present in the group of NPR1-regulated direct target genes, mentioned above. Cysteine-rich repeat proteins have been predicted to be involved in biotic and abiotic stress responses (Venancio and Aravind 2010). For one of the family members, PCC1/PCM4, a role as positive regulator of defense to the biotrophic pathogen *Hyaloperonospora arabidopsidis* has been demonstrated (Sauerbrunn and Schlaich 2004). For another family member, CYSTM3/PCM8, a role as negative regulator of salt stress responses has been reported (Xu et al. 2019). Analysis of Arabidopsis PCM-overexpressing lines revealed that these proteins are positively involved in immunity against pathogens with a (hemi)biotrophic lifestyle. Furthermore, we expanded the potential scope of their function to a role in photomorphogenesis and hypocotyl development.

## Materials and methods

### Plant material and cultivation conditions

*Arabidopsis thaliana* wild-type accession Col-0, mutant *eds1*-*2* (Bartsch et al. 2006), triple mutant *mlo2-5 mlo6*-2 *mlo12*-*1* (Consonni et al. 2006), *hy5 hyh* (Van Gelderen et al. 2018) and PCM-overexpression lines were used in this study. For whole-plant assays with pathogen infection and SA treatment, the seeds were stratified for 48 h in 0.1% agar at 4 °C prior to sowing them on river sand that was saturated with half-strength Hoagland nutrient solution containing 10 μM Sequestreen (Fe-ethylenediamide-di(*0*-hydroxyphenylacetic acid; Ciba-Geigy, Frankfurt, Germany) to supply iron nutrition. After 2 weeks of growing on sand in a closed container which provided 100% relative humidity (RH), the seedlings were transferred to 60-mL pots containing a soil:river sand mixture (12:5, v/v) that had been autoclaved twice for 1 h at 70% RH. Plants were cultivated under a 10-h day (75 μmol m^−2^ s^−1^) and 14-h night cycle at 21 °C. Plants were watered every other day and received modified half-strength Hoagland nutrient solution containing 10 μM Sequestreen once a week. To minimize within-chamber variation, all the trays, each containing a mixture of plant genotype or treatments, were randomized throughout the growth chamber once a week. For the hypocotyl elongation assay, seeds were surface-sterilized and sown on MS plates (8 g L^−1^ agar and 1 g L^−1^ Murashige and Skoog; Duchefa Biochemie B.V., Haarlem, The Netherlands). The seeds were stratified in the dark at 4 °C for 2–3 days before being moved to a climate chamber with long-day conditions (16 h light:8 h dark). After 7 days, the plates were photographed, and hypocotyl length was measured using ImageJ as described previously (De Wit et al. 2016).

The Arabidopsis PCM-overexpression lines were generated by amplifying the coding sequence of genes At2g32190 (*PCM1*), At2g32200 (*PCM2*), At2g32210 (*PCM3*), At3g22231 (*PCM4/PCC1*), At3g22235 (*PCM5*), At3g22240 (*PCM6*), At1g05340 (*PCM7*) and At1g56060 (*PCM8/ ATCYSTM3*) from accession Col-0. The PCM genes were part of a recent paper by Xu et al. (2018) and were named differently in the present study, as clarified in Suppl. Table S1. The primers used for cloning are also listed in Suppl. Table S1. The DNA sequence of the PCR fragments was verified and then cloned using Gateway® cloning (Invitrogen) in the pENTR vector, and subsequently in the pFAST-GO2 Gateway® (Shimada et al. 2010) compatible binary vector under control of the CaMV 35S promoter, followed by sequence verification. Binary vectors were transformed into *Agrabacterium tumefaciens* strain C58C1 containing pGV2260, which was used to transform accession Col-0 with the floral dip method (Clough and Bent 1998). Transformants were selected by growth on ½ MS plates containing DL-Phosphinothricin BASTA, and resistant T_1_ seedlings were transplanted to soil for seed production. T_2_ and T_3_ lines were selected for single insertion of the transgenes using BASTA resistance. Experiments were performed using homozygous T_3_ or T_4_ seeds.

### RNA-seq library preparation and sequencing

The experimental design of the RNA-seq time series experiment with SA-treated Arabidopsis leaves has been described previously (Hickman et al. 2019). In brief, the rosettes of 5-week-old Arabidopsis accession Col-0 plants were dipped into a solution containing 1 mM SA (Mallinckrodt Baker) and 0.015% (v/v) Silwet L77 (Van Meeuwen Chemicals BV), which was added as a surfactant. For mock treatments, plants were dipped into a solution containing 0.015% (v/v) Silwet L77. The sixth leaf (counted from the oldest to the youngest) was harvested from four individual SA- or mock-treated plants at each of the following time points post-treatment: 15 min, 30 min and 1, 1.5, 2, 3, 4, 5, 6, 7, 8, 10, 12 and 16 h. Total RNA was extracted using the RNeasy Mini Kit (Qiagen), including a DNase treatment step in accordance with the manufacturer’s instructions. RNA-seq library preparation and sequencing was performed by UCLA Neuroscience Genomics Core (Los Angeles, CA, USA). Sequencing libraries were prepared using the Illumina TruSeq RNA Sample Prep Kit, and sequenced on the Illumina HiSeq 2000 platform with single read lengths of 50 bases.

For the comparison of the *PCM1-*OX, *PCM5-*OX and *PCM7-*OX lines with wild-type Col-0, two mature leaves (developmental leaf number six and seven) were harvested from two 5-week-old plants per genotype, resulting in two biological replicates. RNA-seq library preparation and sequencing was performed by the Utrecht Sequencing Facility (Utrecht, Netherlands). Sequencing libraries were prepared using the Illumina Truseq mRNA Stranded Sample Prep Kit, and sequenced on the Illumina NextSeq 500 platform with read lengths of 75 bases.

### RNA-seq analysis

Quantification of gene expression from RNA-seq data was performed as described previously (Caarls et al. 2017; Hickman et al. 2017). Reads were mapped to the Arabidopsis genome (TAIR version 10) using TopHat version 2.0.4 (Trapnell et al. 2009) and aligned reads summarized over annotated gene models using HTseq-count (Anders et al. 2015). Genes that were significantly altered over time in response to SA in comparison to the mock treatment were identified using a generalized linear model implemented with the R statistical environment (www.r-project.org). Genes that were differentially expressed between Col-0 and *PCM1-*OX, *PCM5-*OX, or *PCM7-*OX were identified using DESeq2 (Anders and Huber 2010; Love et al. 2014).

### Identification of uncharacterized gene families

Protein sequences of the 630 SA-responsive DEGs with unknown/uncharacterized function (based on gene annotations retrieved from TAIR version 10 (retrieved in 2016) were run through OrthoMCL with default parameters (www.orthomcl.org) (Li et al. 2003). JackHMMER (www.ebi.ac.uk/Tools/hmmer/search/jackhmmer) was then used to identify additional paralogs belonging to the groups identified with OrthoMCL. The phylogentic tree of PCM homologs was generated using PLAZA v4.0 (https://bioinformatics.psb.ugent.be/plaza/) with the *PCM1* gene as a query (Van Bel et al. 2018).

### Determination of transcription factor binding motifs

Transcription factor-gene interactions were inferred from DAP-seq (DNA affinity purification sequencing) experiments, which provide the genome-wide binding profiles of in vitro expressed transcription factors (O’Malley et al. 2016). DAP-seq peaks for 349 Arabidopsis transcription factors with a FRiP (fraction of reads in peaks) score ≥ 5% were retrieved from the Plant Cistrome DB (O'Malley et al. 2016). DAP-seq peaks were used to infer representation of DNA-binding motifs in the promoters of the PCM genes. Motifs are grouped according to cognate transcription factor family.

### Coexpression network analysis

The PCM coexpression network was obtained using the ATTED-II Network Drawer tool with the Ath-r platform (http://atted.jp/cgi-bin/NetworkDrawer.cgi) (Obayashi et al. 2017) using the PCM genes as query genes. Co-expression networks were visualized using Cytoscape v.3.5.1 (Shannon et al. 2003).

### Functional enrichment analysis

GO-term enrichment analysis on gene lists was performed using the GO term finder tool (Boyle et al. 2004). Where indicated, generic GO terms were removed from the analysis by limiting the maximum size of functional categories to 1500 genes.

### Construction of YFP-tagged PCMs and visualization by confocal microscopy

For *in planta* localization experiments, cDNA extracted from Arabidopsis was used to amplify the coding sequences without the stop codon of PCMs using the primers listed in the Suppl. Table S1. The PCR products containing *att*B sequence were cloned into the Gateway pDONR221 vector, then the resulting entry vectors containing the PCM genes were recombined into the Gateway expression vector pB7WGY2, which contains the coding sequence of the Venus fluorescent protein (a variant of yellow fluorescent protein (YFP).

Competent cells of *A. tumefaciens* were transformed with the Gateway expression vector described in the previous paragraph made for protein localization. Transformed colonies were selected using the antibiotic resistance of the vector and rifampicin for which a resistance gene is present in *A. tumefaciens*. Single colonies were grown for 2 days at 28 °C in 20-mL LB medium under shaking conditions. After, the OD_600_ was measured, the cells were pelleted and re-suspended to a final OD_600_ of 0.5 with a ½ MS medium (Duchefa Biochemie) supplemented with 10 mM MES hydrate (Sigma-Aldrich), 20 g L^−1^ sucrose (Sigma-Aldrich), 200 µM acetosyringone (Sigma-Aldrich) at pH 5.6 and incubated in darkness for at least 1 h. The solutions were used to agroinfiltrated the abaxial side of 4–5-week-old *Nicotiana benthamiana* leaves using a 1-mL syringe. The plants were left to grow in normal light conditions and after 2 days leaf sections were taken from agroinfiltrated regions and visualized via confocal microscopy.

Microscopy was performed using a Zeiss LM 700 (Zeiss, Jena, Germany) confocal laser-scanning microscope. Fresh leaf material was prepared on a glass slide with cover slip. Excitation of YFP, plasma membrane FM4-64 dye (Sigma-Aldrich) and autofluorescence of chlorophyll were done at 488 nm. Light emission of YFP was detected at 493–550 nm and the red signal for the FM4-64 dye at 644–800 nm. Analyses of the images were performed with ZEN lite (blue edition).

### Pathogen cultivation and bioassays

*Hyaloperonospora arabidopsidis* isolate Noco2 (*Hpa* Noco2) spores were harvested from infected (*eds1-*2 mutant) plants, eluted through Miracloth, and diluted in water to 50 spores µL^−1^. For the disease bioassay, the leaves of 5-week-old plants were spray-inoculated with this spore suspension. Plants were subsequently placed at 100% RH, under short day conditions (9 h light: 15 h dark) at 16 °C. After 9 days, the spores from eight individual rosette plants were harvested in 5 mL of water and the number of spores per milligram of plant tissue (fresh weight of aerial parts) was counted using a light microscope. Spore counts in the wild-type, mutant and overexpression lines were compared using analysis of variance (ANOVA) followed by Tukey’s multiple comparison tests.

*Pseudomonas syringae* pv. *tomato* (*Pto*) DC3000 was cultured in King’s B medium supplemented with 50 mg L^−1^ rifampicine at 28 °C overnight. Bacteria were collected by centrifugation for 10 min at 5000 *g* and re-suspended in 10 mM MgSO_4_. The suspension was adjusted to OD_600_ = 0.0005 and pressure infiltrated into three mature leaves of 5-week-old plants with a needleless syringe. After 3 days, leaf discs of 5-mm diameter were harvested from two inoculated leaves per plant, representing a single biological replicate. Eight biological replicates were harvested for each genotype. Subsequently, 500 µL of 10 mM MgSO_4_ was added to the leaf discs, after which they were ground thoroughly with metal beads using a TissueLyser (Qiagen). Serial ten-fold dilutions were made in 10 mM MgSO_4_, and 30 µL aliquots plated onto KB agar plates containing 50 mg mL^−1^ rifampicin. After 48 h of incubation at 28 °C, bacterial colonies were counted. Statistical analyses were performed using ANOVA followed by Tukey’s multiple comparison test for means of log_10_-transformed colony counts.

For powdery mildew assays, Arabidopsis plants were inoculated with powdery mildew (*Golovinomyces orontii*) at roughly 2.5 cm rosette size (radius) at 4–5 weeks after germination. *G. orontii* is adapted to infection of Arabidopsis (Kuhn et al. 2016) and was cultivated on susceptible *eds1*-2 plants. Inoculation was conducted by leaf-to-leaf transfer of conidiospores. Leaves from five individual plants were collected at 48 h post inoculation and bleached in 80% ethanol at room temperature for at least 2 days. Prior to microscopic analysis, fungal structures were stained by submerging the leaves in Coomassie staining solution (100% v/v ethanol acid, 0.6% w/v Coomassie blue R-250; Carl Roth, Karlsruhe, Germany) twice for 15–30 s and shortly washed in tap water thereafter. The samples were analyzed with an Axiophot microscope (Carl Zeiss AG). The fungal penetration rate was determined as the percentage of spores successfully developing secondary hyphae over all spores that attempted penetration, visible by an appressorium (Haustorium index). Macroscopic pictures of *G. orontii*-infected plants were taken at 12 days post inoculation with a Coolpix P600 camera (Nikon, Tokyo, Japan). Susceptible Col-0 and the fully resistant *mlo2-5* *mlo6-2* *mlo12-1* triple mutant (Consonni et al. 2006) served as positive and negative controls for infection by *G. orontii*, respectively. Haustorium index in the mutant and overexpression lines were compared using ANOVA followed by Tukey's multiple comparison test.

## Results

### Analysis of uncharacterized SA-responsive genes identifies a family of cysteine-rich transmembrane proteins

**Fig. 1:**
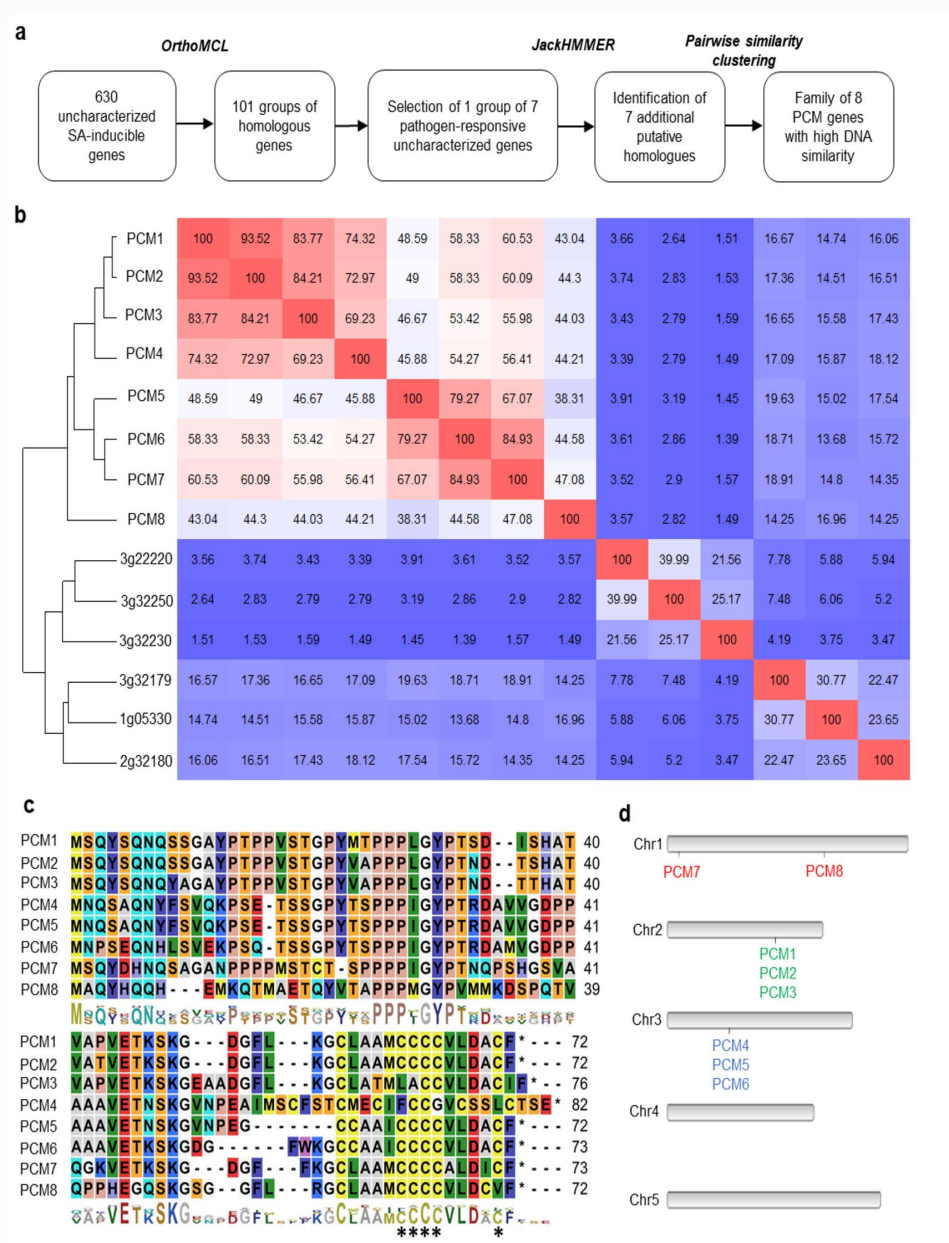
Identification of groups of homologous, uncharacterized SA-inducible genes. Selection of the PCM gene family. **a** Workflow to identify groups of homologous, unknown SA-inducible genes. First, SA-induced DEGs were grouped by DNA similarity using OrthoMCL. One pathogen-responsive group was subjected for further analysis using JackHMMER, followed by pair-wise similarity clustering, revealing a distinct family of eight homologous PCM genes. **b** DNA similarity matrix showing the 14 genes identified by the JackHMMER search. Red and blue indicate high and low similarity, respectively. Unsupervised hierarchical clustering identified a distinct group of PCM genes with high DNA similarity. **c** Amino acid sequence alignment of the eight PCMs. The conserved cysteine-rich transmembrane domain (CYSTM) is highlighted by asterisks. **d** The locations of the eight PCM genes on the Arabidopsis chromosomes (Chr1 to Chr5). The different color gene names reflect PCM gene distribution across chromosomes

**Fig. 2:**
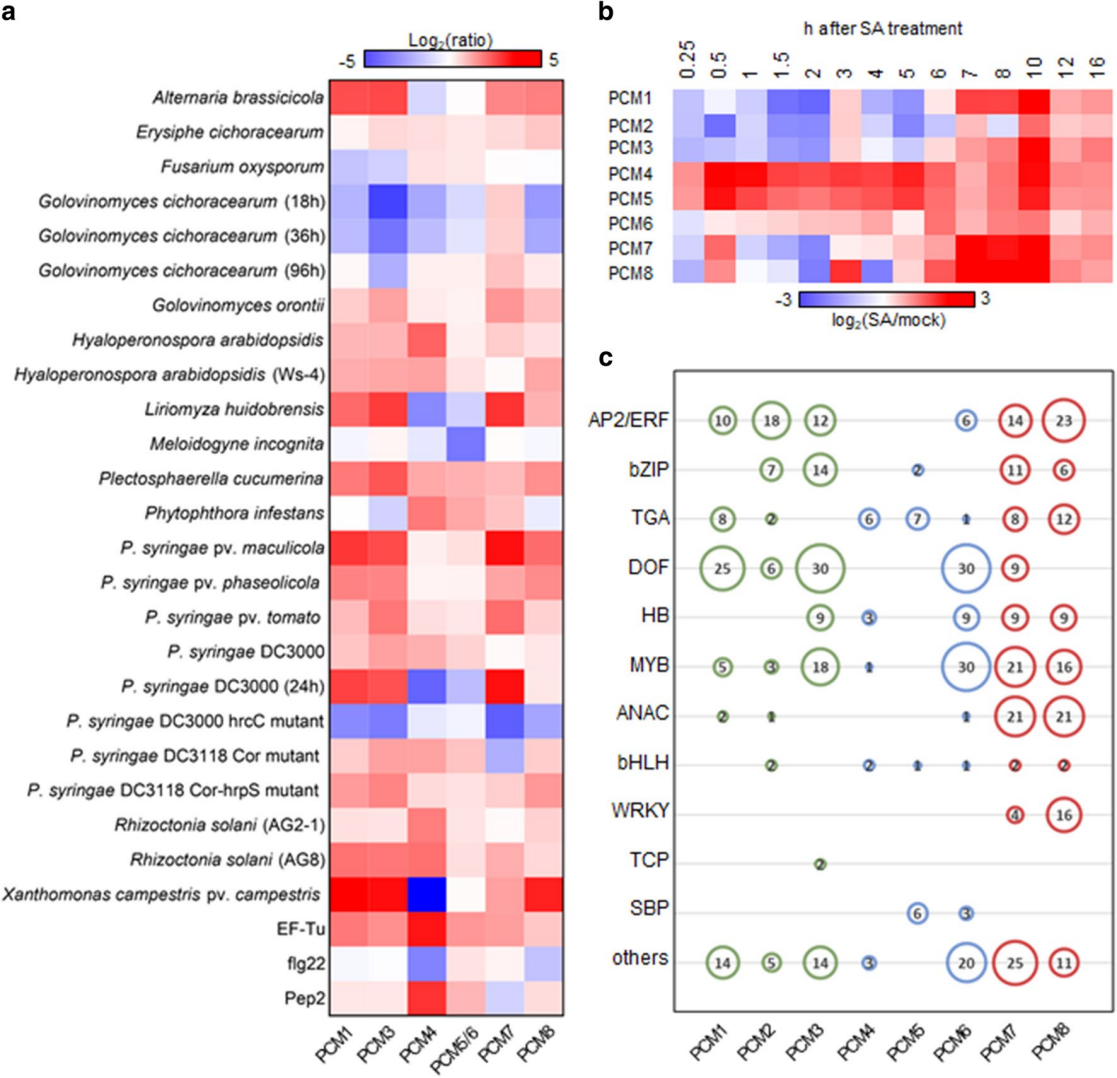
Expression behavior of PCM genes. **a** Genevestigator expression analysis. Shown is a heatmap of expression ratios for the PCM genes following treatments with biotic stressors/elicitors. On the microarrays from which these data are derived (*P* < 0.001) probes for *PCM2* are missing, and the probes for *PCM5* and *PCM6* are shared. Abbreviations that are not explained in the body text: *Ws-4* accession of *Arabidopsis thaliana*, *hrcC* component of the type III secretion system of bacteria, *hrpS* pathogenicity regulatory protein, *Cor* coronatine (a phytotoxin), *AG2-1 and AG8* strains of *Rhizoctonia solani* to which Arabidopsis is susceptible or resistant, respectively, *EF-Tu* bacterial elongation factor (a MAMP), *flg22* 22-amino acid motif of bacterial flagellin (a MAMP), *Pep2* 23-amino acid plant peptide eliciting defenses. **b** Temporal expression of PCM genes over a 16-h time course upon exogenous application of SA. Red and blue indicate increased and decreased expression, respectively. **c** Representation of DNA-binding motifs in the promoters of the PCM genes. Motifs are grouped according to cognate transcription factor family. The size and number in each circle represent the number of transcription factors per-family that bind to a certain DNA motif present in the PCM genes, according to the DAP-seq data of O’Malley et al. (2016)

Recently, we used high-throughput RNA-seq analysis to profile genome-wide changes in mRNA abundance in Arabidopsis leaves following treatment with SA over a 16-h period. Analysis of these transcriptome data identified 9524 genes that were differentially expressed between mock- and SA-treated leaves (Hickman et al. 2019). Subsequent investigation of functional annotations associated with these differentially expressed genes (DEGs) revealed that 630 of these genes encode proteins of unknown or uncharacterized function. Because of the central role of SA in defense against pathogen infection, we hypothesized that among these genes would be genes with undiscovered roles in plant immunity. To simplify the analysis and functional interpretation of these uncharacterized genes, we first divided them in groups based on amino acid sequence similarity. To achieve this, we used OrthoMCL (Li et al. 2003), which is a tool for identifying homologous relationships between sets of proteins. This analysis resulted in a division of 101 groups of putative homologs, each comprising between two and nine members (Suppl. Data Set S1; Fig. 1a). Because we were specifically interested in genes that are involved in defense against pathogens, we analyzed gene expression behavior, using available gene expression data from Genevestigator (http://www.genevestigator.ethz.ch/) (Hruz et al. 2008). This pointed to a group of seven genes that were highly induced by a variety of immune elicitors and pathogens (Fig. 2a) and that were responsive to SA in our RNA-seq experiment (Fig. 2b). Their increased transcription was confirmed by an independent experiment in which genome-wide transcriptional changes as induced by exogenous SA treatment were analyzed using microarrays (Suppl. Fig. S2; Pajerowska-Mukhtar et al. 2012).

To identify all possible paralogs (including remote paralogs), the seven genes were used as queries in JackHMMER (Finn et al. 2015) (www.ebi.ac.uk/Tools/hmmer/search/jackhmmer). JackHMMER is a highly sensitive homology detection tool that can identify shared protein domains among matched sequences, as defined according to Pfam domains (Finn et al. 2015). This analysis led to the prediction of seven additional paralogs (Fig. 1b). Next, we quantified the degree of nucleotide sequence identity between the 14 proteins by constructing a nucleotide sequence identity matrix (Fig. 1b), which was followed by unsupervised clustering of the similarity matrix, leading to the identification of a distinct family of eight small genes (< 82 amino acids) with high nucleotide sequence identity (> 38%). All of the seven originally selected genes of unassigned function belong to this group, but also included now *PCC1* (*PCM4* in Fig. 1), which has a reported role in defense and is regulated by the circadian clock (Sauerbrunn and Schlaich 2004). One other member, *CYSTM3* (*PCM8* in Fig. 1), has very recently also been characterized and shown to negatively influence salt stress resistance (Xu et al. 2019). Suppl. Table S1 lists all of the PCM genes with their Arabidopsis Genome Initiative (AGI) number and alternative name. The genes in this family all encode short proteins (71–82 amino acids) with a conserved cysteine-rich transmembrane (CYSTM) domain, as predicted by the JackHMMER analyses (Fig. 1c, CYSTM domain indicated by asterisks). To reflect their regulation and enrichment for cysteine residues in the encoded proteins, this eight-member gene family was named Pathogen-induced Cysteine-rich transMembrane proteins (PCMs). The PCM gene family contains two distinct gene clusters; the *PCM1*, *PCM2* and *PCM3* genes are situated in tandem on Arabidopsis chromosome 2, while *PCM4* (*PCC1*), *PCM5* and *PCM6* are tandemly arrayed on chromosome 3 (Fig. 1d). Furthermore, *PCM7* and *PCM8* (*CYSTM3*) are positioned at distant locations on chromosome 1. The expression behavior of the eight PCM genes is broadly along the lines of the three subgroups, showing overlap but also differences with members of the other subgroups (Fig. 2a and b). This is in accordance with varying overrepresentation of different transcription factor binding DNA motifs in the promoters of the eight PCM genes (Fig. 2c). The remainder of this paper explores the significance of the PCM protein family and its three subgroups in plant immunity and development.

### Subcellular localization of PCMs

**Fig. 3:**
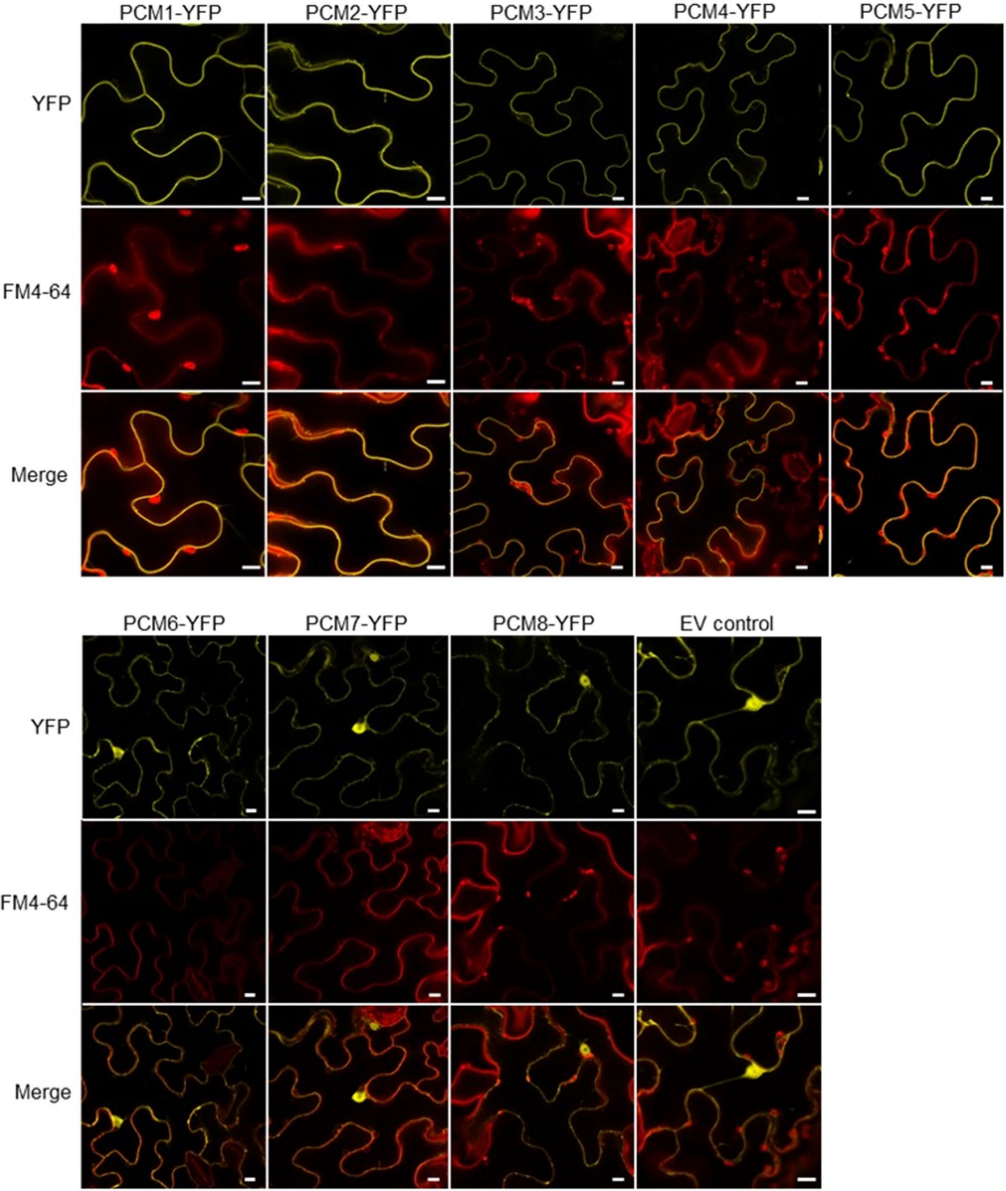
Subcellular localization of PCM-YFP fusion proteins**. **Confocal images of transiently transformed *N. benthamiana* epidermal leaf cells expressing the eight YFP-tagged PCM proteins under control of the CaMV 35S promoter. Representative fluorescence images are shown of PCM-YFP or free YFP (control by expression of the empty vector, EV) in the top panels, of FM 4‐64 labelling of the membranes in the middle panels, and of the overlay of YFP and FM 4-64 in the bottom panels. Bar = 10 μm

The characteristic CYSTM domain that resides in the PCM protein family is encoded by a total of 98 genes across 33 plant species (Suppl. Fig. S1). Transmembrane domains enable protein functions across membranes (Sharpe et al. 2010; Luschnig and Vert 2014) and are often conserved across kingdoms when the respective protein has a specialized function (e.g., photoreceptors in eyes of mammals and insects) (Fischer et al. 2004). To begin to characterize the PCMs, we determined their subcellular localization by fusing Venus YFP to the C-terminus of all eight PCM proteins and expressing these fusion proteins under the control of the constitutively active cauliflower mosaic virus (CaMV) 35S promoter. Expression of the empty vector (EV-YFP, resulting in free YFP) served as a control. The dye FM 4-64 was used as a membrane marker. The fusion proteins were transiently expressed in *N. benthamiana* leaves by infiltration with *A. tumefaciens* carrying the different constructs. Confocal microscopy analysis showed that for five of the PCM fusion proteins, namely PCM1, PCM2, PCM3, PCM4 and PCM5, the YFP signal overlapped with the fluorescent signal of the plasma membrane-localized FM 4-64 dye (Fig. 3). This suggests that these PCM proteins are plasma membrane localized. By contrast, in case of YFP-tagged PCM6, PCM7 and PCM8, the YFP signals were detected prevalently in the cytoplasm and the nucleus (Fig. 3), which could either be indicative of a non-membrane localization of these proteins or reflect undesired cleavage of the YFP label.

### PCM coexpression analysis points to specificity in PCM function

**Fig. 4:**
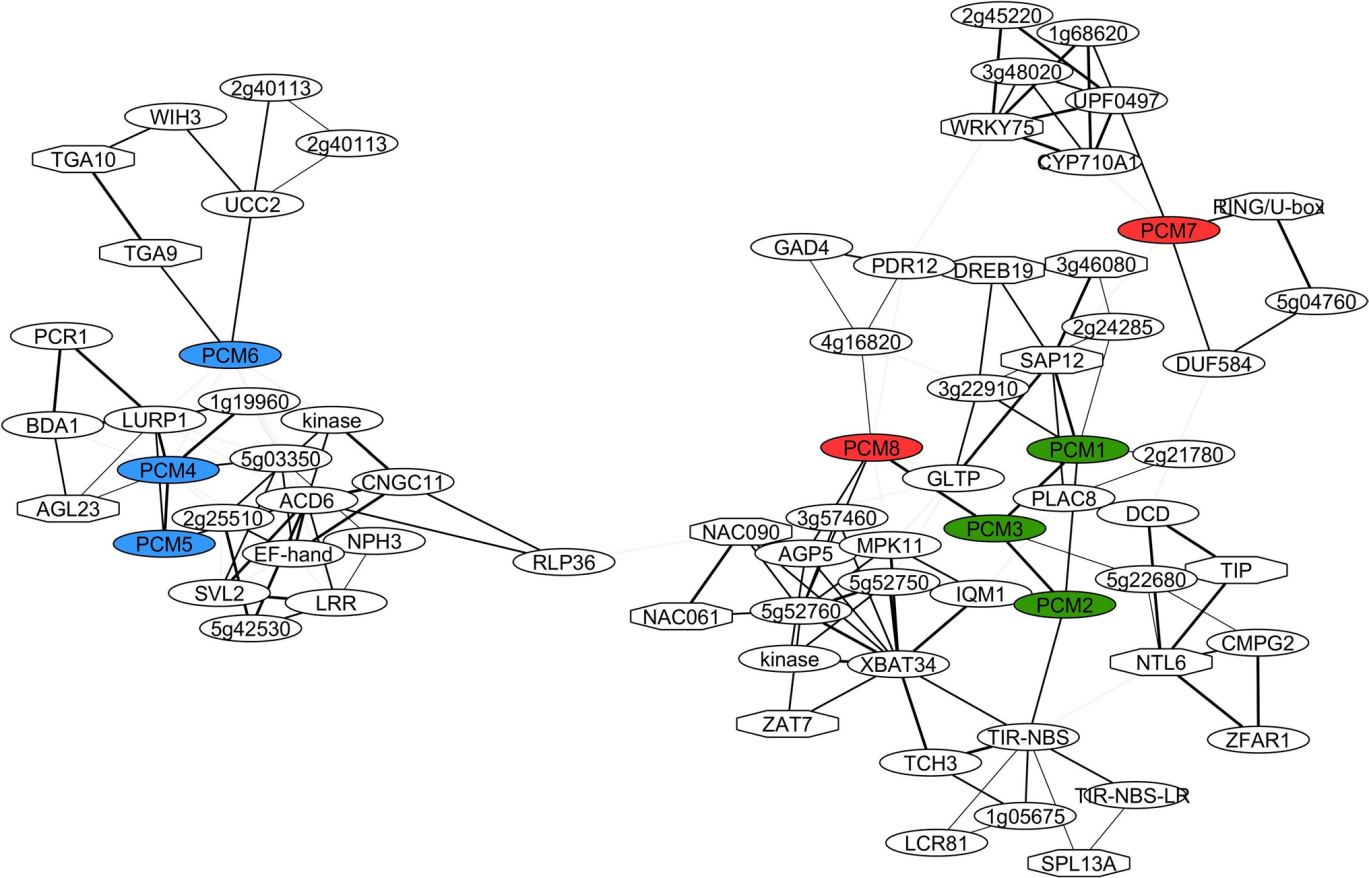
PCM coexpression networks. Coexpression network obtained using the ATTED-II Network Drawer tool on whole-genome transcriptome data sets with the PCM genes as bait. Hexagonal-shaped nodes indicate genes encoding transcriptional regulators. The thickness of the lines is proportional to the extent of coexpression of the linked gene

Because genes with related biological functions often have similar expression patterns, a well-established method to investigate gene function is the construction and analysis of gene coexpression networks (Vandepoele et al. 2009). Using the eight PCM genes as query we generated PCM coexpression networks using publicly available microarray and RNA-seq datasets with the ATTED-II coexpression tool (Obayashi et al. 2017) (Fig. 4). The PCM coexpression network was enriched for genes associated with defense responses (*P* < 0.01; hypergeometric test) and included known defense-related genes, such as *LURP1*, *ACD6*, *RLP36*, *NTL6*, *NAC61*, *NAC90*, *ZFAR1*, *PDR12*, *WRKY75* and *MPK11,* suggesting a role for the PCM protein family in plant defense. Within the PCM coexpression network, coexpression neighborhoods of members of the three PCM subgroups (Fig. 2) overlap. Interestingly, the coexpression neighborhood occupied by subgroup II (*PCM4*, *PCM5* and *PCM6*) was distinct from that of all other PCM genes. Also, *PCM7* was part of a relatively isolated coexpression subnetwork. On the other hand, *PCM8* shared its coexpression neighborhood to a large extent with that of subgroup I (*PCM1*, *PCM2* and *PCM3*). In sum, our coexpression network analysis suggests a role for all eight PCM genes in plant defense, but also highlights subnetworks, suggesting functional diversification and/or differential regulation of the PCM subgroups. This notion is further supported by the distinct gene expression behavior of the different PCM subgroups after treatment with pathogens or exogenous SA (Fig. 2a, b, and Suppl. Fig. S2) and the presence of different transcription factor binding sites in the promoters of the PCM genes (Fig. 2c).

### PCM-overexpressing lines show enhanced resistance to (hemi-)biotrophic pathogens

**Fig. 5:**
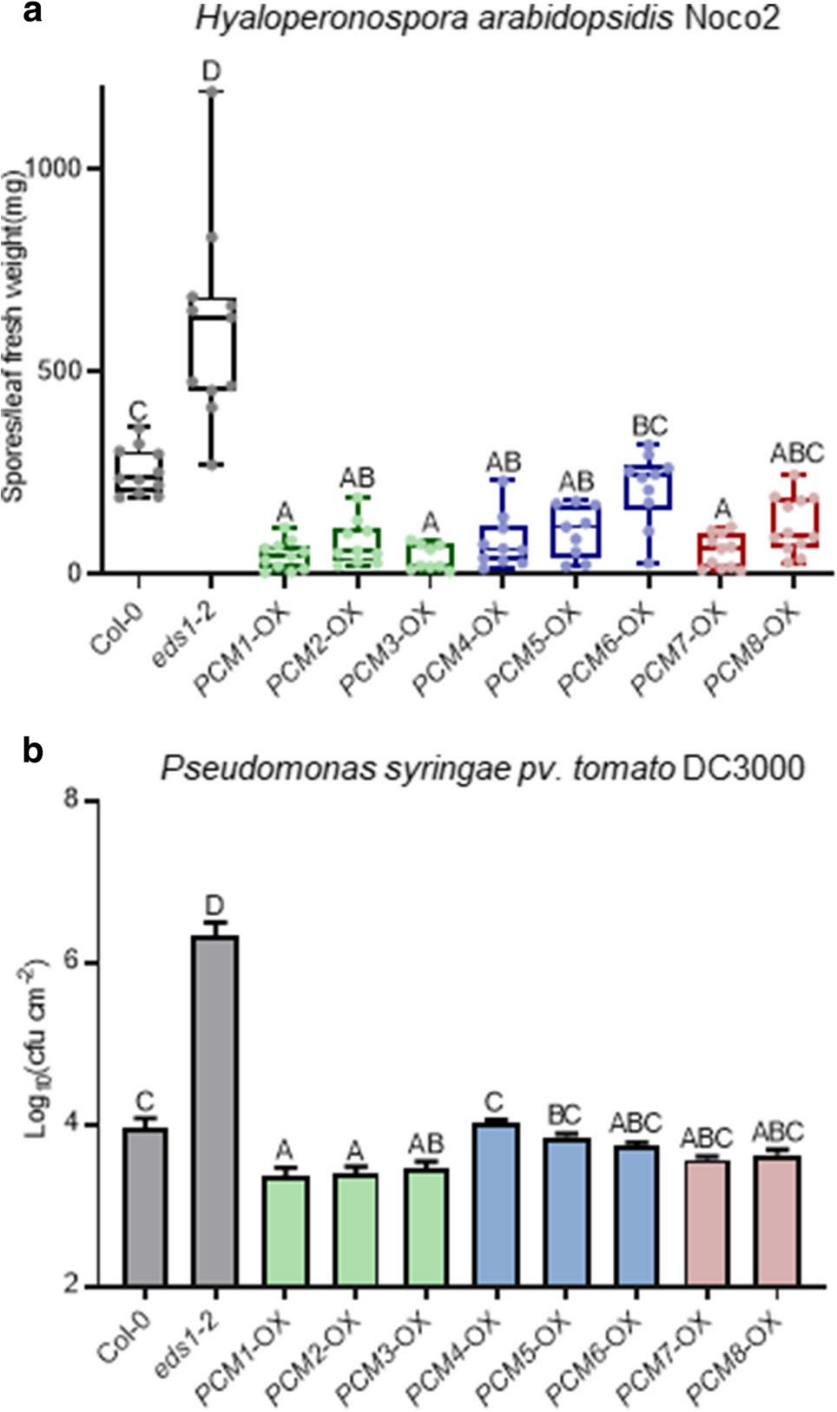
Overexpression of PCMs enhances resistance to *Hyaloperonospora arabidopsidis* Noco2 and *Pseudomonas syringae* pv. *tomato* DC3000. **a** Quantification of *Hpa* Noco2 sporulation on 5-week-old wild-type (Col-0), *eds1*-2 and transgenic lines constitutively overexpressing individual PCM genes under the control of the CaMV 35S promoter (PCM-OX) at 10 days post inoculation (dpi) by spraying (*n* = 9–12*).* **b** Bacterial multiplication of *Pto* DC3000 in wild-type (Col-0), *eds1*-2 and PCM-OX lines at 3 dpi by pressure infiltration (*n* = 8). Means ± SE (error bars) are shown. Letters denote significant differences between genotypes (one-way ANOVA, Tukey’s post-hoc test, *P* < 0.05)

To investigate the hypothesis that members of the PCM protein family play a role in plant immunity, transgenic Arabidopsis lines expressing the individual PCM genes under the control of the CaMV 35S promoter were generated. The transgenic PCM-overexpression (PCM-OX) lines were of unaltered size and did not show any obvious developmental abnormalities (Suppl. Fig. S3). RNA-seq analysis (see below) confirmed the overexpression status of the PCM-OX lines for genes *PCM1* and *PCM7*, but not for *PCM5* whose overexpression levels might have remained below the thresholds of statistical analysis (Suppl. Data Set S2). Because the PCM gene family responded to exogenous SA treatment (Fig. 2b and Suppl. Fig. S2), the PCM-OX lines were screened for an altered level of resistance to two pathogens that are controlled by SA-dependent defenses: the obligate biotrophic oomycete *H. arabidopsidis* Noco2 (*Hpa* Noco2) and the hemi-biotrophic bacterium *P. syringae* pv. *tomato* DC3000 (*Pto* DC3000). For both assays, the performance of 5-week-old PCM-OX lines was compared to that of the wild-type (Col-0) and the super susceptible mutant *eds1*-2 of the same age. Apart from *PCM6*, overexpression of all other PCM genes led to reduced *Hpa* Noco2 spore formation when compared to wild-type plants (Fig. 5a). *Pto* DC3000 propagation was significantly decreased in the *PCM1*-OX, *PCM2-*OX, and *PCM3-*OX lines but not in the other lines (Fig. 5b). These findings suggest that the vast majority of PCM family members is positively involved in host defense against *Hpa* Noco2*,* while a protective effect against *Pto* DC3000 is only evident for the subgroup I of the PCM protein family comprising PCM1, PCM2, and PCM3.

### Transcriptome analysis of PCM-OX lines reveals no upregulation of typical immune responses

**Fig. 6:**
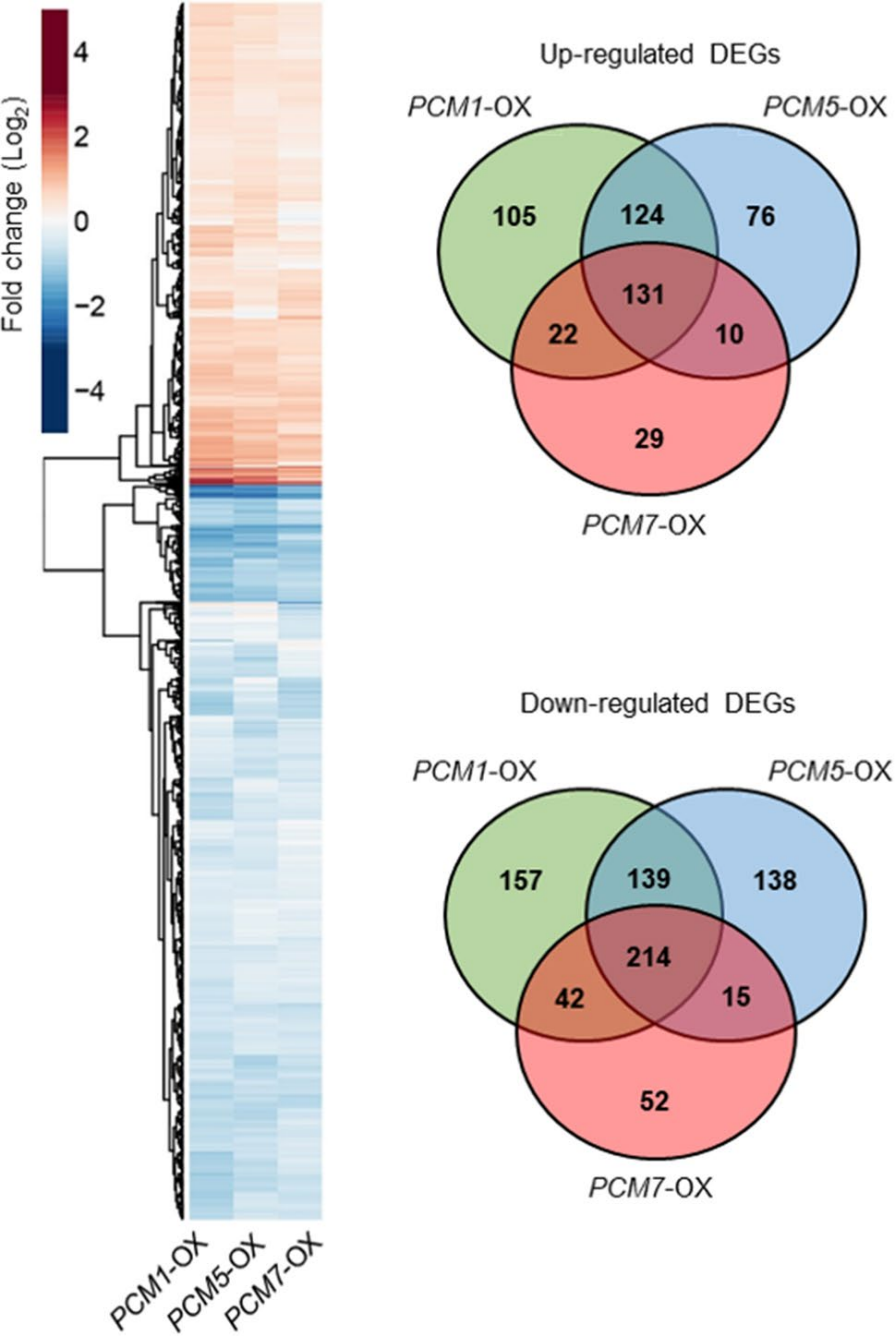
Transcriptome analysis of PCM-OX lines. **a** Heatmap (left) showing up- and downregulation of genes in the *PCM1*-OX, *PCM5*-OX or *PCM7*-OX lines in comparison to wild-type Col-0 plants, as revealed by RNA-seq analysis. **b** Venn diagrams (right) indicating the overlap between DEGs in each of the PCM-OX lines

To gain insight into the mechanisms underlying the enhanced disease resistance phenotype obtained by overexpression of the PCM genes, we analyzed the transcriptome of three PCM-OX lines, each representing a member of the three PCM subgroups: *PCM1*-OX (subgroup I), *PCM5*-OX (subgroup II), and *PCM7*-OX (subgroup III). These PCM members of the respective subgroups were representing the small differences between the different subgroups based on DNA and amino acid composition (Fig. 1b and c), chromosome position (Fig. 1d), induction by pathogens (Fig. 2a) and SA (Fig. 2b), enrichment in transcription factor binding motifs (Fig. 2c), inclusion in gene coexpression networks (Fig. 4), and effects on disease resistance (Fig. 5). RNA-seq analysis was performed on leaf tissue harvested from 5-week-old, non-treated plants. Differential expression analysis revealed that in the *PCM1-*OX, *PCM5-*OX and *PCM7-*OX lines 934, 873, and 515 genes, respectively, were differentially expressed in comparison to wild-type Col-0 plants (*P* < 0.05*,* fold change > 2) (Suppl. Data Set S2). Among the DEGs there were *PCM1* in the *PCM1*-OX line and *PCM7* in the *PCM7*-OX line, each showing a twofold log increase in transcript abundance. Notably, the list of DEGs comprised no other PCM gene in any of the overexpression lines, indicating that there is no compensatory regulation of other family members in this situation. There was considerable overlap between the expression profiles of the three PCM*-*OX lines (Fig. 6a). Of all DEGs, 27% were upregulated or downregulated in all three lines (in the same direction), whereas 44% were specifically up- or downregulated in a single overexpression line (Fig. 6b). More genes were downregulated (60%) than upregulated (40%).

**Fig. 7:**
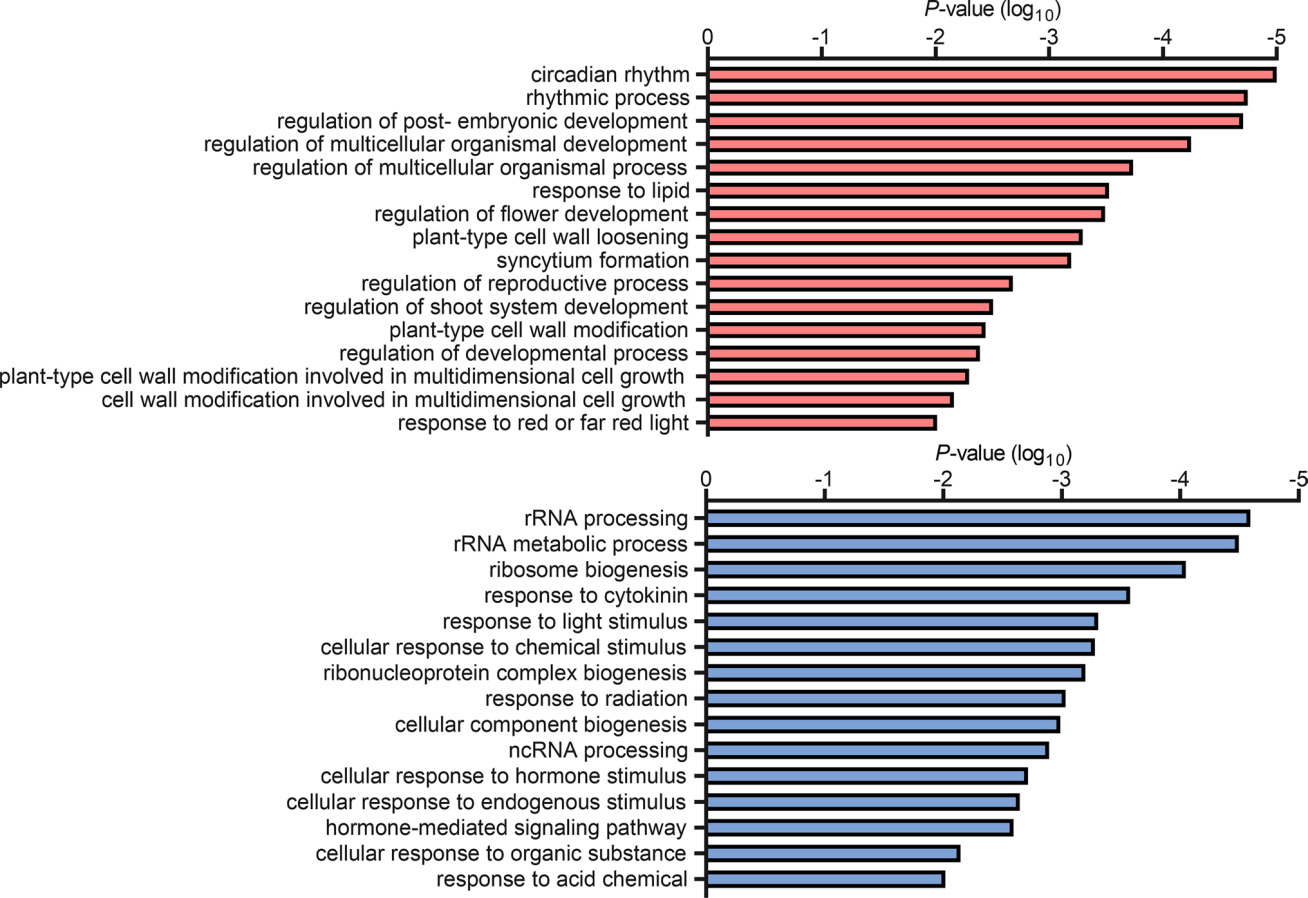
GO terms enriched among genes up- or downregulated in PCM-OX lines. Shown are the GO terms significantly enriched among the genes that are significantly upregulated (top) or downregulated (bottom) in *PCM1*-OX, *PCM5*-OX and *PCM7*-OX, when compared to wild type

The overlapping 131 upregulated DEGs shared by all three PCM*-*OX lines were not enriched for typical immunity-related functions (Fig. 7 and Suppl. Data Set S3). Instead, the term ‘circadian rhythm’ was the most significantly enriched specific category, with additional enriched terms including ‘regulation of multicellular organismal development’ ‘plant cell wall loosening’, and ‘response to red or far-red light’. The shared 214 downregulated DEGs by all three PCM*-*OX lines were associated with functional categories, such as ‘rRNA processing’, ‘response to cytokinin’ and ‘response to light stimulus’ (Fig. 7 and Suppl. Data Set S3). There was also no enrichment of purely immunity-related categories DEGs that were specifically up- or downregulated in any of the *PCM1-*OX, *PCM5-*OX or *PCM7-*OX lines (Suppl. Data Sets S2 and S3). However, general terms like ‘response to hormone’ were overrepresented in up- and/or downregulated DEGs of different lines. ‘Glucosinolate process’ was detected particularly in the upregulated DEGs of the *PCM7*-OX line, while ‘nucleolus’ was enriched in both the up- and downregulated DEGs of *PCM7*-OX, as well as in downregulated DEGs of *PCM5*-OX (Suppl. Data Set S3). Overall, based on these data, we hypothesize that pathogen-induced PCM production contributes to an increased level of defense through an impact on developmental processes in the cells that may affect pathogen performance.

### Involvement of PCMs in hypocotyl elongation

**Fig. 8:**
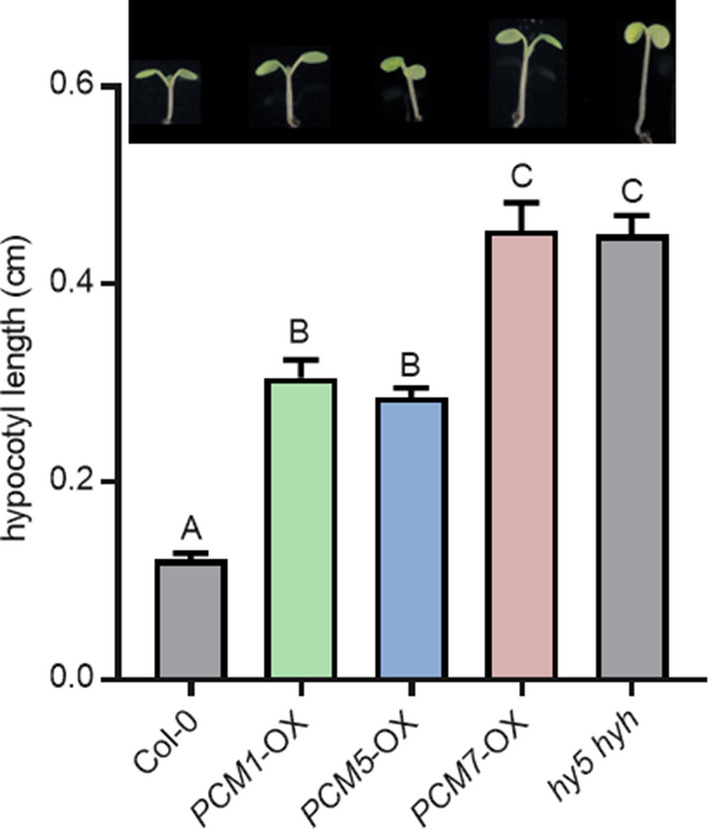
PCM1, PCM5, and PCM7 influence hypocotyl elongation. Hypocotyl lengths of 7-day-old Col-0, *PCM1*-OX, *PCM5*-OX, *PCM7*-OX and *hy5 hyh* seedlings grown in vitro in white light (*n* = 20). Means ± SE (error bars) are shown. Letters denote significant differences between genotypes (one-way ANOVA, Tukey’s post-hoc test, *P* < 0.05). Inset: representative pictures of 7-day-old seedlings. The experiment was repeated twice with similar results

There was no clear link to plant immunity among the genes differentially expressed in the three PCM-OX lines assayed, while the association with developmental processes and light responses was obvious (Fig. 7). In all three PCM*-*OX lines, the *HY5* and *HYH* genes, which are master regulators of light signaling (Van Gelderen et al. 2018) and also respond to pathogen infection (Genevestigator data) were upregulated (Suppl. Data Set S2). This prompted us to investigate morphogenic responsiveness of the PCM-OX lines. In shade-avoiding plants, such as Arabidopsis, perception of far-red light triggers morphological adaptations, such as elongation of the hypocotyl and petioles, to reach for better quality light (Ballaré 2014). The *hy5 hyh* double mutant, which is affected in HY5 and its closely related HY5 homolog (HYH), displays such elongated hypocotyl growth compared to wild-type plants when cultivated in white light (Van Gelderen et al. 2018). Unexpectedly, the hypocotyl length of the *PCM1-*OX*, PCM5-*OX*,* and *PCM7*-OX lines was also greater than that of the wild type, and the hypocotyl of *PCM7*-OX was even of the same size as that of the positive control, the *hy5 hyh* double mutant (Fig. 8). This points to a role for PCMs in modulating both growth and development. Possibly, the PCMs affect HY5 protein activity or stability, which is compensated by an enhanced expression level of the *HY5* gene. Altogether, our data suggest dual roles for PCMs in defense and in photomorphogenesis.

## Discussion

Despite more than two decades of research efforts focused on the model plant Arabidopsis, a significant fraction (over 13%) of genes found in this plant are not characterized to any extent (Luhua et al. 2013; Niehaus et al. 2015). Our analysis of SA-responsive genes in Arabidopsis leaves revealed that 630 genes encode proteins of unknown function. Using a protein homology search, we grouped these uncharacterized genes into 101 groups of paralogs that likely encode proteins with similar functions (Fig. 1a; Suppl. Data Set S1). We validated whether such an approach could aid the functional annotation of groups of unknown genes. Therefore, we selected and further characterized a family of eight pathogen-induced cysteine-rich transmembrane proteins (PCMs). The PCM genes formed three subgroups, based on their nucleotide similarity, amino acid similarity, and chromosomal position (Fig. 1b–d). The expression profiles of the PCM members under different biotic stress conditions and SA treatment broadly followed that of the three subgroups, showing some overlap but also differences between the subgroups (Figs. 2a and b). This is in agreement with the commonalities and dissimilarities in transcription factor-binding sites detected in the promotor regions of the eight PCM genes (Fig. 2c), and the overlap or isolation of the coexpression networks of the PCM genes (Fig. 4).

Given the complexity of the PCM family and its overlap in coexpressed genes, we expected functional redundancy between members and thus resorted to using overexpression lines rather than knockout mutants for the functional analysis of this protein family. Overexpression of one PCM member of each of the three subgroups (*PCM1*-OX, *PCM5*-OX and *PCM7*-OX) revealed 27% overlap of all DEGs in all three lines, and 44% of all DEGs were specifically regulated in only one of the lines (Fig. 6). A function for the PCMs in defense was evidenced by the enhanced resistance of PCM-overexpression lines to the biotrophic pathogens *Hpa* Noco2 and *Pto* DC3000 (Fig. 5). Moreover, the overexpression of PCMs resulted in differential expression of genes related to light and development (Fig. 7, Suppl. Data Sets S2 and S3), and elongated hypocotyl growth of seedlings (Fig. 8), suggesting an additional role for PCMs in photomorphogenesis. Though we only used single PCM-OX lines in our sets of experiments, the shared phenotypes regarding DEGs, pathogen resistance and photomorphogenesis of the different tested PCM-OX lines suggest that the detected effects are authentic consequences of PCM function and do not result from unintentional chromosomal rearrangements at the location where the transgenes (PCMs) were inserted.

### Membrane association of CYSTM domain-containing PCMs

The PCMs are small proteins (< 84 amino acids) that contain a predicted cysteine-rich transmembrane C-terminus domain (CYSTM), which is a rare domain, but highly conserved among eukaryotic organisms. CYSTM domain-containing proteins are present in diverse species, including Arabidopsis, *Caenorhabditis elegans*, *Candida albicans*, *Homo sapiens*, *Mus musculus*, *Oryza sativa*, *Saccharomysces cerevisae* and *Zea mays* (Venancio and Aravind 2010)*.* The molecular mechanism by which the CYSTM module functions is not clear yet, but the proteins appear to play a role in stress tolerance, for example, by altering the redox potential of membranes, thereby quenching radical species to protect the plant, or by affecting membrane-associated protein functions (Kuramata et al. 2009; Venancio and Aravind 2010).

The conserved cysteines may serve to interact with a ligand, e.g. other PCMs, which could result in homo- or heterodimerization as shown in yeast expression systems for several Arabidopsis PCM family members (Mir and León 2014; Xu et al. 2018). However, the PCMs can potentially also interact with other protein partners, as shown for PCM4/PCC1, which interacts with its N-terminal part (cytoplasm-faced, non-CYSTM containing) with the subunit 5 of the COP9 signalosome at the plasma membrane. This may lead to post-translational control of multiple protein targets involved in diverse biological processes, such as light signaling, development, and immunity (Mir et al. 2013; Mir and León 2014).

We experimentally confirmed a tight association of PCM1-PCM5 with the cell periphery and the fluorescent FM4-64 marker (Fig. 3), suggesting that these proteins are anchored to the plasma membrane. This localization could potentially promote a change in local lipid composition, as shown for PCM4/PCC1 (Mir et al. 2013), and also affect the membrane structure. This notion is supported by changes in gene expression observed in the PCM-OX lines, which highlighted enrichment in GO terms related to ‘response to lipid’, cell wall modification’, and ‘regulation of development’ (Fig. 7). Moreover, membrane alterations may block the invasion of intracellular pathogens like *Hpa* (Fig. 5a) that form an intricate interface with the host membrane. There may also be consequences for membrane permeability or activity of (defense regulatory) proteins associated with the plasma membrane. While plasma membrane localization of PCM1-PCM5 was supported by experiments using YFP-tagged PCMs in transiently transformed *N. benthamiana* leaves, this was not the case for PCM6-PCM8 (Fig. 3). The latter finding is consistent with a recent study by Xu et al. (2018) who also found cytoplasmic localization of these proteins using the same study system. However, these authors also reported cytoplasmic localization for PCM1, PCM2, PCM3 and PCM5, for which we detected solely plasma membrane localization, which is in line with the expectations based on the presence of the CYSTM domain (Venancio and Aravind 2010) and early reports on PCC1/PCM4 (Mir and León 2014). At this point, we cannot exclude that the nucleo-cytoplasmic localization of PCM6-PCM8 detected by us and others (Xu et al. 2018) is due to degradation of the PCM-YFP fusion protein in this experimental setup. Alternative experimental approaches, such as biochemical analyses, will thus be required to corroborate the subcellular localization of these proteins.

### The function of CYSTM domain-containing PCMs in plant defense

The *PCM4* gene is also known as *PCC1* and has previously been identified as an early-activated gene upon infection with the bacterial pathogen *Pto* carrying the avirulence gene AvrRpt2 and to be controlled by the circadian clock (Sauerbrunn and Schlaich 2004). Microarray analysis of *npr1*-*1* plants revealed that in addition to several pathogenesis-related (*PR*) genes, the expressions of *PCC1/PCM4* and *PCM6* were affected in this mutant (Wang et al. 2006). Later, *PCC1/PCM4* was identified to be induced by UV-C light in an SA-dependent manner, potentially playing a role as activator of stress-stimulated flowering in Arabidopsis (Segarra et al. 2010). Transgenic plants carrying the *β-glucuronidase* (*GUS*) reporter gene showed that the expression of *PCC1/PCM4* in the seedling stage was confined to the root vasculature and the stomatal guard cells of cotyledons, but spread to the petioles and the whole limb of fully expanded leaves (Mir et al. 2013). *PCC1/PCM4*-OX lines showed enhanced resistance to *Hpa* (Sauerbrunn and Schlaich 2004; Fig. 5a), while RNAi plants were more susceptible to the hemi-biotrophic oomycete pathogen *Phytophthora brassicae* and more resistant to the necrotrophic fungal pathogen *Botrytis cinerea* when compared with wild-type plants (Mir et al. 2013). We confirmed that *PCC1*/*PCM4*-overexpression lines are resistant to *Hpa* and extend this finding to additional PCMs: Overexpression of *PCM1*, *PCM2*, *PCM3*, *PCM5*, *PCM7*, and *PCM8* also provided protection against *Hpa* infection (Fig. 5a). This points to a common underlying defense mechanism that is activated by the PCMs, which might be related to an altered membrane environment as we discussed in the previous paragraph. This mechanism may also be responsible for the enhanced protection against *Pto* infection that we observed by overexpressing *PCM1*, *PCM2*, and *PCM3* (Fig. 5b). The lack of effect on *Pto* of the other PCM-OX lines, however, also points to divergent effects of the different *PCMs*, which is corroborated by the partly distinct DEG sets of the *PCM1*-, *PCM5*-, and *PCM7*-OX lines (Fig. 6b). We also assayed the *PCM1*, *PCM5* and *PCM7* overexpressors for resistance to the biotrophic powdery mildew fungus *Golovinomyces orontii* but found that these lines displayed the same level of disease development (haustorium formation and macroscopic symptoms) as the wild type, whereas the triple mutant *mlo2 mlo6 mlo12*, serving as a positive control, was highly resistant (Suppl. Fig. S4). It may be that the protection mechanism provided by the PCMs is not effective against this pathogen species, but it may also be that the strain that was used in the bioassays was so virulent that it could have overcome any quantitative resistance accomplished by PCM overexpression.

Notably, cultivated PCM-OX lines did not show any morphological abnormalities, such as dwarfism, and the RNA-seq data of the *PCM1*-, *PCM5*-, and *PCM7*-OX lines did not reveal any evidence for the constitutive expression of typical defense-related genes (such as PR genes) that would explain the enhanced disease resistance of these plant. In the future, it will be of interest to elucidate the yet unrecognized mechanisms that contribute to this phenotype. Conditioned by the antagonistic interplay of defense-associated phytohormones (Leon-Reyes et al. 2010), plants with enhanced resistance to biotrophic pathogens often show enhanced susceptibility to necrotrophic pathogens. For instance, infection with hemi-biotrophic *Pseudomonas syringae*, which induces SA-mediated defense, rendered plants more susceptible to the necrotrophic pathogen *Alternaria brassicicola* by suppression of the jasmonate (JA) signaling pathway (Spoel et al. 2007). It will thus be also interesting to explore how the PCM-OX lines perform upon challenge with necrotrophic pathogens.

### Interplay between immunity and photomorphogenesis

Our transcriptome data revealed that the *PCM1*-, *PCM5*-, and *PCM7*-OX lines were primarily enriched for genes and biological functions related to circadian rhythm, light signaling, and growth and development (Fig. 7 and Suppl. Data Set S2). The *PCM4*/*PCC1* gene had previously been reported to respond to circadian rhythm and UV-C light, and to influence stress-induced flowering (Segarra et al. 2010). Here, we show that the *PCM1*-, *PCM5*-, and *PCM7*-OX lines exhibit elongated hypocotyl growth compared to wild-type plants (Fig. 8). This phenotype is shared with the *hy5 hyh* double mutant, suggesting that the PCMs promote photomorphogenesis. Several studies have addressed the connection between plant defense and light signaling; e.g. UV-C induces SA-dependent defenses, and high levels of far-red light (as in shade) repress defense responses to both pathogen and insects, as reviewed by Ballaré (2014). A recent paper by Nozue et al. (2018) reported that SA pathway genes are key components of shade avoidance; *PCM4* and *PCM5* were part of the downregulated gene set by high far-red levels, and showed an altered expression level in shade avoidance syndrome mutants. Therefore, a double role for the PCMs in defense and photomorphogenesis is not unexpected. How the PCMs accomplish this dual function is not clear yet. Like discussed earlier, the PCMs might influence membrane structure and activity of proteins that reside in the membrane or that bind to PCMs, like the subunit 5 of the COP9 signalosome (Mir et al. 2013; Mir and León 2014). These diverse effects may independently influence defense and photomorphogenesis, but an interdependence between the two biological processes, where one is a consequence of the other, is also a possibility.

In conclusion, our approach led to the identification of the family of PCM proteins that carry the distinctive CYSTM module, and which have a broad biological impact on plant performance, as shown by enhanced protection against biotrophic pathogens and increased hypocotyl growth in PCM-OX lines. We elucidated some molecular effects of the PCMs by showing that the majority of the PCM members localize to the plasma membrane, that the PCM genes are responsive to SA and pathogen challenge, and that overexpression of PCMs leads to the induction of genes associated with light responses and development, but not to typical defense-associated responses.

#### *Author contribution statement*

MCVV and SCMVW conceived and designed the research. MPM, RH, MCVV, NMN, and AR conducted the experiments. MPM, RH, RP and MCVV analyzed the data. MPM, RH, CMJP and SCMVW wrote the manuscript. All authors read and approved the manuscript.

## Supplementary Information

Below is the link to the electronic supplementary material.

Supplementary file1 (DOCX 867 KB)

Supplementary file2 (PPTX 45 KB)

Supplementary file3 (XLSX 23 KB)

Supplementary file4 (XLSX 224 KB)

Supplementary file5 (XLSX 48 KB)

## Abbreviations

CYSTM: Cysteine-rich transmembrane
DEGs: Differentially expressed genes
GO: Gene ontology
*Hpa*: *Hyaloperonospora arabidopsidis*
NPR1: NONEXPRESSOR OF PATHOGENESIS-RELATED GENES1
PCM: Pathogen-induced cysteine-rich transmembrane protein
*Pto*: *Pseudomonas syringae* pv. *tomato*
SA: Salicylic acid
YFP: Yellow fluorescent protein

## References

[CR1] Anders S, Huber W (2010). Differential expression analysis for sequence count data. Genome Biol.

[CR2] Anders S, Pyl PT, Huber W (2015). HTSeq—a Python framework to work with high-throughput sequencing data. Bioinformatics.

[CR3] Arabidopsis Genome Initiative (2000). Analysis of the genome sequence of the flowering plant *Arabidopsis thaliana*. Nature.

[CR4] Ballaré CL (2014). Light regulation of plant defense. Annu Rev Plant Biol.

[CR5] Bar-Joseph Z, Gitter A, Simon I (2012). Studying and modelling dynamic biological processes using time-series gene expression data. Nat Rev Genet.

[CR6] Bartsch M, Gobbato E, Bednarek P, Debey S, Schultze JL, Bautor J, Parker JE (2006). Salicylic acid-independent ENHANCED DISEASE SUSCEPTIBILITY1 signaling in *Arabidopsis* immunity and cell death is regulated by the monooxygenase *FMO1* and the Nudix hydrolase *NUDT7*. Plant Cell.

[CR7] Boller T, Felix G (2009). A renaissance of elicitors: perception of microbe-associated molecular patterns and danger signals by pattern-recognition receptors. Annu Rev Plant Biol.

[CR8] Boyle EI, Weng SA, Gollub J, Jin H, Botstein D, Cherry JM, Sherlock G (2004). GO::TermFinder—open source software for accessing Gene Ontology information and finding significantly enriched Gene Ontology terms associated with a list of genes. Bioinformatics.

[CR9] Breeze E, Harrison E, McHattie S, Hughes L, Hickman R, Hill C, Kiddle S, Kim YS, Penfold CA, Jenkins D, Zhang CJ, Morris K, Jenner C, Jackson S, Thomas B, Tabrett A, Legaie R, Moore JD, Wild DL, Ott S, Rand D, Beynon J, Denby K, Mead A, Buchanan-Wollaston V (2011). High-resolution temporal profiling of transcripts during *Arabidopsis* leaf senescence reveals a distinct chronology of processes and regulation. Plant Cell.

[CR10] Caarls L, Van der Does D, Hickman R, Jansen W, Van Verk MC, Proietti S, Lorenzo O, Solano R, Pieterse CMJ, Van Wees SCM (2017). Assessing the role of ETHYLENE RESPONSE FACTOR transcriptional repressors in salicylic acid-mediated suppression of jasmonic acid-responsive genes. Plant Cell Physiol.

[CR11] Clough SJ, Bent AF (1998). Floral dip: a simplified method for *Agrobacterium*-mediated transformation of *Arabidopsis thaliana*. Plant J.

[CR12] Consonni C, Humphry ME, Hartmann HA, Livaja M, Durner J, Westphal L, Vogel J, Lipka V, Kemmerling B, Schulze-Lefert P, Somerville SC, Panstruga R (2006). Conserved requirement for a plant host cell protein in powdery mildew pathogenesis. Nature Genet.

[CR13] Coolen S, Proietti S, Hickman R, Davila Olivas NH, Huang PP, Van Verk MC, Van Pelt JA, Wittenberg AH, De Vos M, Prins M, Van Loon JJ, Aarts MG, Dicke M, Pieterse CMJ, Van Wees SCM (2016). Transcriptome dynamics of Arabidopsis during sequential biotic and abiotic stresses. Plant J.

[CR14] De Wit M, Keuskamp DH, Bongers FJ, Hornitschek P, Gommers CMM, Reinen E, Martinez-Ceron C, Fankhauser C, Pierik R (2016). Integration of phytochrome and cryptochrome signals determines plant growth during competition for light. Curr Biol.

[CR15] Dodds PN, Rathjen JP (2010). Plant immunity: towards an integrated view of plant-pathogen interactions. Nat Rev Genet.

[CR16] Finn RD, Clements J, Arndt W, Miller BL, Wheeler TJ, Schreiber F, Bateman A, Eddy SR (2015). HMMER web server: 2015 update. Nucleic Acids Res.

[CR17] Fischer JA, Acosta S, Kenny A, Cater C, Robinson C, Hook J (2004). Drosophila klarsicht has distinct subcellular localization domains for nuclear envelope and microtubule localization in the eye. Genetics.

[CR18] Fu ZQ, Dong X (2013). Systemic acquired resistance: turning local infection into global defense. Annu Rev Plant Biol.

[CR19] Gamir J, Darwiche R, Van't Hof P, Choudhary V, Stumpe M, Schneiter R, Mauch F (2017). The sterol-binding activity of PATHOGENESIS-RELATED PROTEIN 1 reveals the mode of action of an antimicrobial protein. Plant J.

[CR20] Hayat Q, Hayat S, Irfan M, Ahmad A (2010). Effect of exogenous salicylic acid under changing environment: a review. Environ Exp Bot.

[CR21] Hickman R, Van Verk MC, Van Dijken AJH, Pereira Mendes M, Vroegop-Vos IA, Caarls L, Steenbergen M, Van der Nagel I, Wesselink GJ, Jironkin A, Talbot A, Rhodes J, De Vries M, Schuurink RC, Denby K, Pieterse CMJ, Van Wees SCM (2017). Architecture and dynamics of the jasmonic acid gene regulatory network. Plant Cell.

[CR22] Hickman R, Pereira Mendes M, Van Verk MC, Van Dijken AJH, Di Sora J, Denby K, Pieterse CMJ, Van Wees SCM (2019). Transcriptional dynamics of the salicylic acid response and its interplay with the jasmonic acid pathway. BioRxiv.

[CR23] Hruz T, Laule O, Szabo G, Wessendorp F, Bleuler S, Oertle L, Widmayer P, Gruissem W, Zimmermann P (2008). Genevestigator V3: a reference expression database for the meta-analysis of transcriptomes. Adv Bioinformatics.

[CR24] Krouk G, Mirowski P, LeCun Y, Shasha DE, Coruzzi GM (2010). Predictive network modeling of the high-resolution dynamic plant transcriptome in response to nitrate. Genome Biol.

[CR25] Kuhn H, Kwaaitaal M, Kusch S, Acevedo-Garcia J, Wu H, Panstruga R (2016). Biotrophy at its best: novel findings and unsolved mysteries of the Arabidopsis-powdery mildew pathosystem. Arabidopsis Book.

[CR26] Kuramata M, Masuya S, Takahashi Y, Kitagawa E, Inoue C, Ishikawa S, Youssefian S, Kusano T (2009). Novel cysteine-rich peptides from *Digitaria ciliaris* and *Oryza sativa* enhance tolerance to cadmium by limiting its cellular accumulation. Plant Cell Physiol.

[CR27] Leon-Reyes A, Van der Does D, De Lange ES, Delker C, Wasternack C, Van Wees SCM, Ritsema T, Pieterse CMJ (2010). Salicylate-mediated suppression of jasmonate-responsive gene expression in Arabidopsis is targeted downstream of the jasmonate biosynthesis pathway. Planta.

[CR28] Lewis LA, Polanski K, De Torres-Zabala M, Jayaraman S, Bowden L, Moore J, Penfold CA, Jenkins DJ, Hill C, Baxter L, Kulasekaran S, Truman W, Littlejohn G, Prusinska J, Mead A, Steinbrenner J, Hickman R, Rand D, Wild DL, Ott S, Buchanan-Wollaston V, Smirnoff N, Beynon J, Denby K, Grant M (2015). Transcriptional dynamics driving MAMP-triggered immunity and pathogen effector-mediated immunosuppression in Arabidopsis leaves following infection with *Pseudomonas syringae* pv *tomato* DC3000. Plant Cell.

[CR29] Li L, Stoeckert CJ, Roos DS (2003). OrthoMCL: identification of ortholog groups for eukaryotic genomes. Genome Res.

[CR30] Love MI, Huber W, Anders S (2014). Moderated estimation of fold change and dispersion for RNA-seq data with DESeq2. Genome Biol.

[CR31] Luhua S, Hegie A, Suzuki N, Shulaev E, Luo X, Cenariu D, Ma V, Kao S, Lim J, Gunay MB, Oosumi T, Lee SC, Harper J, Cushman J, Gollery M, Girke T, Bailey-Serres J, Stevenson RA, Zhu JK, Mittler R (2013). Linking genes of unknown function with abiotic stress responses by high-throughput phenotype screening. Physiol Plant.

[CR32] Luna E, Pastor V, Robert J, Flors V, Mauch-Mani B, Ton J (2011). Callose deposition: a multifaceted plant defense response. Mol Plant-Microbe Interact.

[CR33] Luschnig C, Vert G (2014). The dynamics of plant plasma membrane proteins: PINs and beyond. Development.

[CR34] Martínez C, Pons E, Prats G, León J (2004). Salicylic acid regulates flowering time and links defence responses and reproductive development. Plant J.

[CR35] Mir R, León J (2014). Pathogen and circadian controlled 1 (PCC1) protein is anchored to the plasma membrane and interacts with subunit 5 of COP9 signalosome in *Arabidopsis*. PLoS ONE.

[CR36] Mir R, Hernández ML, Abou-Mansour E, Martínez-Rivas JM, Mauch F, Métraux JP, León J (2013). Pathogen and Circadian Controlled 1 (PCC1) regulates polar lipid content, ABA-related responses, and pathogen defence in *Arabidopsis thaliana*. J Exp Bot.

[CR37] Niehaus TD, Thamm AM, De Crecy-Lagard V, Hanson AD (2015). Proteins of unknown biochemical function: a persistent problem and a roadmap to help overcome it. Plant Physiol.

[CR38] Nozue K, Devisetty UK, Lekkala S, Mueller-Moule P, Bak A, Casteel CL, Maloof JN (2018). Network analysis reveals a role for salicylic acid pathway components in shade avoidance. Plant Physiol.

[CR39] Obayashi T, Aoki Y, Tadaka S, Kagaya Y, Kinoshita K (2017). ATTED-II in 2018: a plant coexpression database based on investigation of the statistical property of the mutual rank index. Plant Cell Physiol.

[CR40] O'Malley RC, Huang SC, Song L, Lewsey MG, Bartlett A, Nery JR, Galli M, Gallavotti A, Ecker JR (2016). Cistrome and epicistrome features shape the regulatory DNA landscape. Cell.

[CR41] Pajerowska-Mukhtar KM, Wang W, Tada Y, Oka N, Tucker CL, Fonseca JP, Dong X (2012). The HSF-like transcription factor TBF1 is a major molecular switch for plant growth-to-defense transition. Curr Biol.

[CR42] Rivas-San Vicente M, Plasencia J (2011). Salicylic acid beyond defence: its role in plant growth and development. J Exp Bot.

[CR43] Sauerbrunn N, Schlaich NL (2004). *PCC1*: a merging point for pathogen defence and circadian signalling in *Arabidopsis*. Planta.

[CR44] Segarra S, Mir R, Martinez C, León J (2010). Genome-wide analyses of the transcriptomes of salicylic acid-deficient versus wild-type plants uncover Pathogen and Circadian Controlled 1 (PCC1) as a regulator of flowering time in Arabidopsis. Plant Cell Environ.

[CR45] Sels J, Mathys J, De Coninck BM, Cammue BP, De Bolle MF (2008). Plant pathogenesis-related (PR) proteins: a focus on PR peptides. Plant Physiol Biochem.

[CR46] Shannon P, Markiel A, Ozier O, Baliga NS, Wang JT, Ramage D, Amin N, Schwikowski B, Ideker T (2003). Cytoscape: a software environment for integrated models of biomolecular interaction networks. Genome Res.

[CR47] Sharpe HJ, Stevens TJ, Munro S (2010). A comprehensive comparison of transmembrane domains reveals organelle-specific properties. Cell.

[CR48] Shimada TL, Shimada T, Hara-Nishimura I (2010). A rapid and non-destructive screenable marker, FAST, for identifying transformed seeds of *Arabidopsis thaliana*. Plant J.

[CR49] Spoel SH, Johnson JS, Dong X (2007). Regulation of tradeoffs between plant defenses against pathogens with different lifestyles. Proc Natl Acad Sci USA.

[CR50] Trapnell C, Pachter L, Salzberg SL (2009). TopHat: discovering splice junctions with RNA-Seq. Bioinformatics.

[CR51] Tsuda K, Somssich IE (2015). Transcriptional networks in plant immunity. New Phytol.

[CR52] Van Bel M, Diels T, Vancaester E, Kreft L, Botzki A, Van de Peer Y, Coppens F, Vandepoele K (2018). PLAZA 4.0: an integrative resource for functional, evolutionary and comparative plant genomics. Nucleic Acids Res.

[CR53] Van Gelderen K, Kang C, Paalman R, Keuskamp D, Hayes S, Pierik R (2018). Far-red light detection in the shoot regulates lateral root development through the HY5 transcription factor. Plant Cell.

[CR54] Van Loon LC, Rep M, Pieterse CMJ (2006). Significance of inducible defense-related proteins in infected plants. Annu Rev Phytopathol.

[CR55] Vandepoele K, Quimbaya M, Casneuf T, De Veylder L, Van de Peer Y (2009). Unraveling transcriptional control in Arabidopsis using *cis*-regulatory elements and coexpression networks. Plant Physiol.

[CR56] Venancio TM, Aravind L (2010). CYSTM, a novel cysteine-rich transmembrane module with a role in stress tolerance across eukaryotes. Bioinformatics.

[CR57] Wang D, Amornsiripanitch N, Dong X (2006). A genomic approach to identify regulatory nodes in the transcriptional network of systemic acquired resistance in plants. PLoS Pathog.

[CR58] Windram O, Madhou P, McHattie S, Hill C, Hickman R, Cooke E, Jenkins DJ, Penfold CA, Baxter L, Breeze E, Kiddle SJ, Rhodes J, Atwell S, Kliebenstein DJ, Kim YS, Stegle O, Borgwardt K, Zhang CJ, Tabrett A, Legaie R, Moore J, Finkenstadt B, Wild DL, Mead A, Rand D, Beynon J, Ott S, Buchanan-Wollaston V, Denby KJ (2012). Arabidopsis defense against *Botrytis cinerea*: Chronology and regulation deciphered by high-resolution temporal transcriptomic analysis. Plant Cell.

[CR59] Xu Y, Yu Z, Zhang D, Huang J, Wu C, Yang G, Yan K, Zhang S, Zheng C (2018). CYSTM, a novel non-secreted cysteine-rich peptide family, involved in environmental stresses in *Arabidopsis thaliana*. Plant Cell Physiol.

[CR60] Xu Y, Yu Z, Zhang S, Wu C, Yang G, Yan K, Zheng C, Huang J (2019). CYSTM3 negatively regulates salt stress tolerance in *Arabidopsis*. Plant Mol Biol.

