# Supplementary material for: A family of pathogen-induced cysteine-rich transmembrane proteins is involved in plant disease resistance
Source: Planta. 2021 Apr 15;253(5):102. doi: 10.1007/s00425-021-03606-3 (PMC8049917; doi:10.1007/s00425-021-03606-3)
Supplement: Supplementary file 1 — Supplementary file1 (DOCX 867 KB) [file 425_2021_3606_MOESM1_ESM.docx]

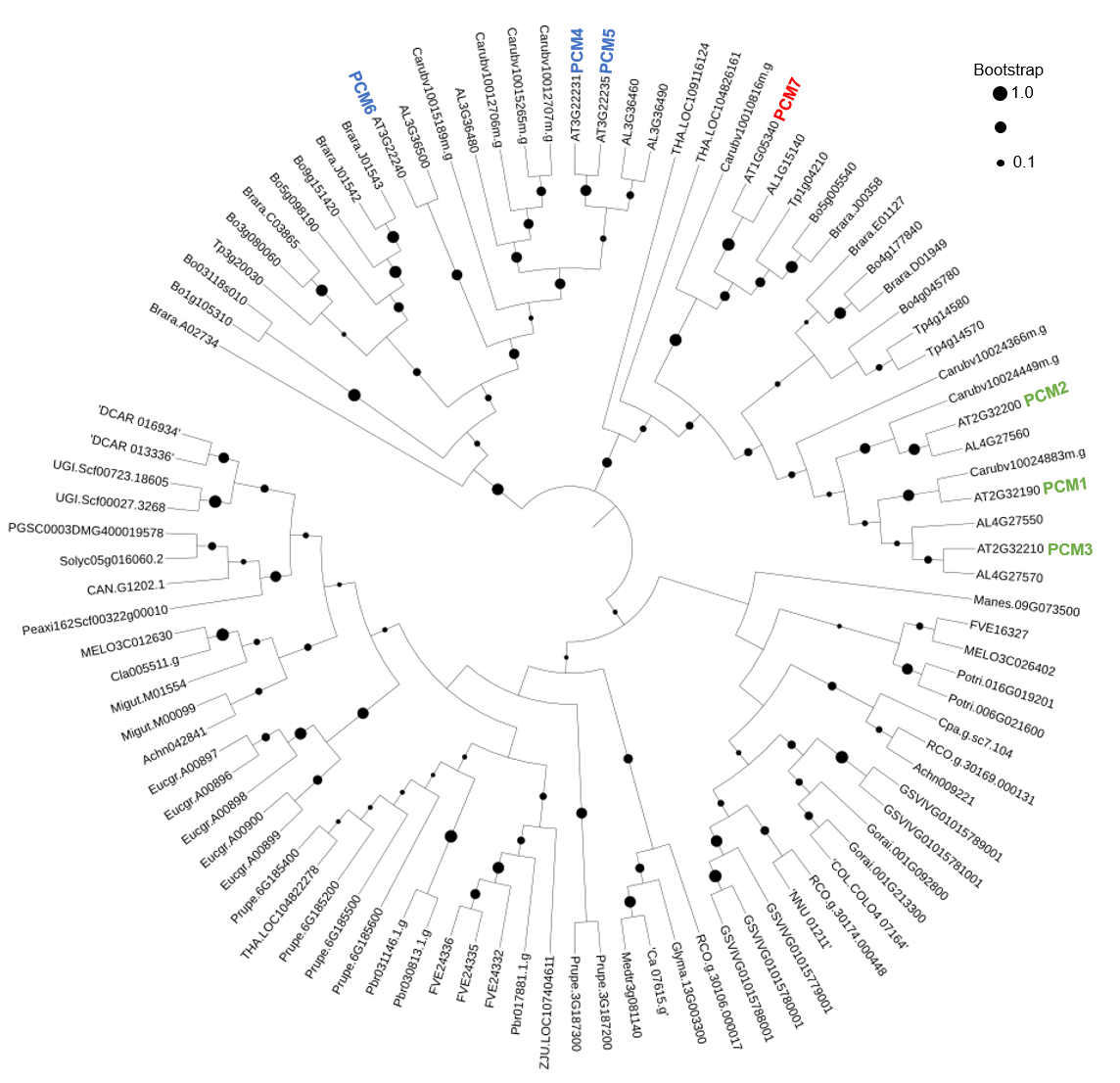


**Supplemental Fig. S1** Phylogenetic relationship of closely related homologs of the PCM gene family in Arabidopsis and 32 other plant species. The PLAZA platform included the isoform of PCM8 in which the CYSTM domain is excised, which is the reason why PCM8 is not included in this phylogenetic tree. Differently sized black dots indicate bootstrap support according to the legend on the top right


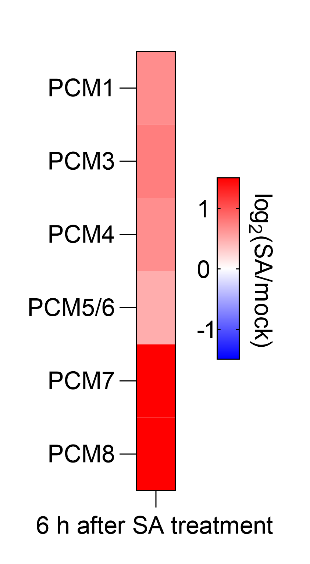


**Supplemental Fig. S2** Expression analysis of PCM genes at 6 h after exogenous application of SA. Shown is a heatmap of the expression ratio of SA/mock based on microarray data that are reported by Pajerowska-Mukhtar et al. (2012). Probes for *PCM2* are missing, and the probes for *PCM5* and *PCM6* are shared. Different shades of red indicate different degrees of increased expression


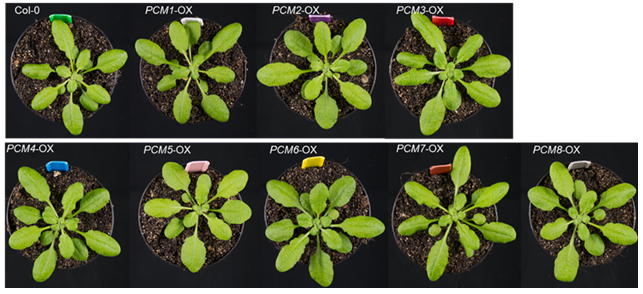


**Supplemental Fig. S3** Growth of PCM-OX lines. Representative photos of 5-week-old plants for each genotype


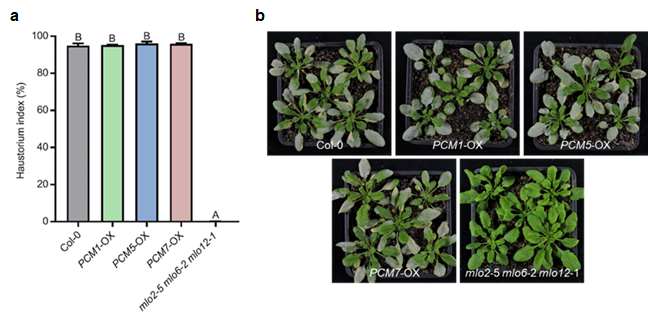


**Supplemental Fig. S4** Powdery mildew (*G. orontii*) infection phenotypes of PCM-OX lines. **a** Quantitative analysis of host cell entry (at 48 h post inoculation) on wild-type Col-0, the fully resistant *mlo2*-5 *mlo6*-2 *mlo12*-1 triple mutant and *PCM1*-OX, *PCM5*-OX and *PCM7*-OX lines (*n* = 5). Letters denote significant differences between genotypes (one-way ANOVA, Tukey’s post-hoc test, *P* < 0.05). **b** Macroscopic infection phenotypes of the same lines as shown in **a** at 12 days post inoculation

**Supplemental Table S1.** List of PCM genes, their AGI numbers (ID) and alternative names. Primer sequences used for cloning

|  | ID | Other name | Forward and reverse primers (5’ – 3’) |
| --- | --- | --- | --- |
| *PCM1* | AT2G32190 | *ATCYSTM4* | F: ATGAGCCAATACAGCCAAAACCAATCTTC  R: GAAGCAGGCGTCGAGGACACAA |
| *PCM2* | AT2G32200 | *ATCYSTM5* | F: ATGAGCCAATACAGTCAAAACCAATATGCAG  R: GAAAATGCATGCGTCGAGGACGCAA |
| *PCM3* | AT2G32210 | *ATCYSTM6* | F: ATGAGTCAATACAGCCAAAACCAATCTTCAG  R: GAAGCATGCGTCGAGGACACAACAA |
| *PCM4* | AT3G22231 | *PCC1* | F: ATGAATCAATCCGCGCAAAATTACTTTTCCG  R: CTCTGATGTACAGAGGCTGGAGCAT |
| *PCM5* | AT3G22235 | *ATCYSTM8* | F: ATGAATCAATCCGCGCAAAATTACTTTTCCG  R: GAAGCATGCATCCAGGACACAACAG |
| *PCM6* | AT3G22240 | *ATCYSTM9* | F: ATGAATCCATCCGAGCAGAATCACTTGTC  R: GAAGCATGCATCCAGGACACAACAG |
| *PCM7* | AT1G05340 | *ATCYSTM1* | F: ATGAGCCAGTACGATCACAACCAGTC  R: GAAGCAAATGTCCAGGGCACAACAG |
| *PCM8* | AT1G56060 | *ATCYSTM3* | F: ATGGCTCAGTATCATCAACAGCATGAAATG  R: GAAGACACAATCCAAAACGCAGCAGC |
