# Supplementary material for: A family of pathogen-induced cysteine-rich transmembrane proteins is involved in plant disease resistance
Source: Planta. 2021 Apr 15;253(5):102. doi: 10.1007/s00425-021-03606-3 (PMC8049917; doi:10.1007/s00425-021-03606-3)
Supplement: Supplementary file 2 — Supplementary file2 (PPTX 45 KB) [file 425_2021_3606_MOESM2_ESM.pptx]

## Slide 1
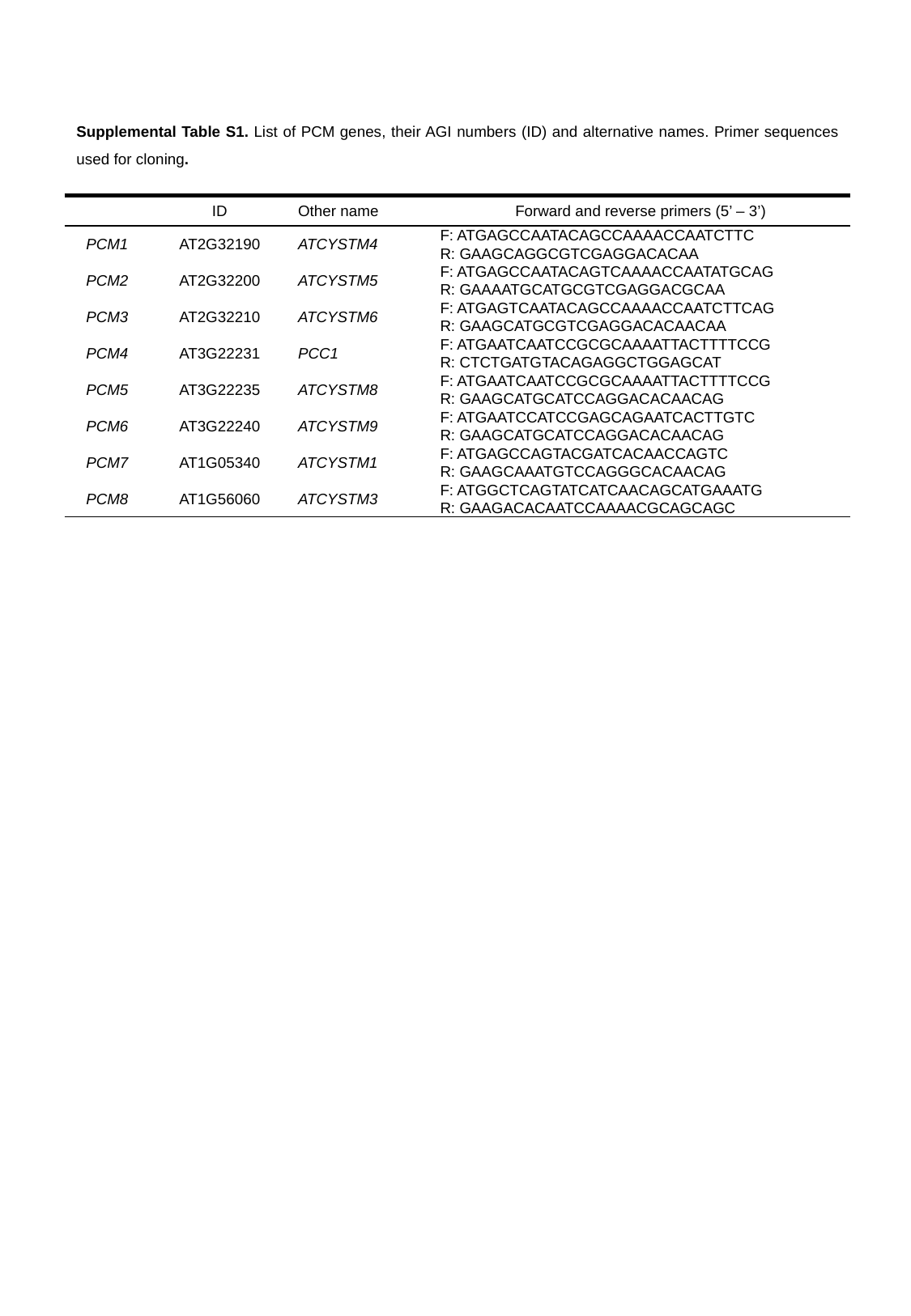

Supplemental Table S1. List of PCM genes, their AGI numbers (ID) and alternative names. Primer sequences used for cloning.
| | ID | Other name | Forward and reverse primers (5’ – 3’) |
| --- | --- | --- | --- |
| PCM1 | AT2G32190 | ATCYSTM4 | F: ATGAGCCAATACAGCCAAAACCAATCTTC R: GAAGCAGGCGTCGAGGACACAA |
| PCM2 | AT2G32200 | ATCYSTM5 | F: ATGAGCCAATACAGTCAAAACCAATATGCAG R: GAAAATGCATGCGTCGAGGACGCAA |
| PCM3 | AT2G32210 | ATCYSTM6 | F: ATGAGTCAATACAGCCAAAACCAATCTTCAG R: GAAGCATGCGTCGAGGACACAACAA |
| PCM4 | AT3G22231 | PCC1 | F: ATGAATCAATCCGCGCAAAATTACTTTTCCG R: CTCTGATGTACAGAGGCTGGAGCAT |
| PCM5 | AT3G22235 | ATCYSTM8 | F: ATGAATCAATCCGCGCAAAATTACTTTTCCG R: GAAGCATGCATCCAGGACACAACAG |
| PCM6 | AT3G22240 | ATCYSTM9 | F: ATGAATCCATCCGAGCAGAATCACTTGTC R: GAAGCATGCATCCAGGACACAACAG |
| PCM7 | AT1G05340 | ATCYSTM1 | F: ATGAGCCAGTACGATCACAACCAGTC R: GAAGCAAATGTCCAGGGCACAACAG |
| PCM8 | AT1G56060 | ATCYSTM3 | F: ATGGCTCAGTATCATCAACAGCATGAAATG R: GAAGACACAATCCAAAACGCAGCAGC |
